# High-Resolution Mapping of DTP1–3 and MCV1 Immunization Coverage and Estimates of Zero-Dose and Under-Vaccinated Children in the Democratic Republic of the Congo

**DOI:** 10.3390/vaccines14090754

**Published:** 2026-08-29

**Authors:** Krishnaveni K. Sasi, Chigozie E. Utazi, Heather R. Chamberlain, A. Cunningham, Pierre Z. Akilimali, Attila N. Lazar, Andrew J. Tatem

**Affiliations:** 1 WorldPop, School of Geography and Environmental Science, University of Southampton, Southampton SO17 1BJ, UK; c.e.utazi@soton.ac.uk (C.E.U.); a.j.tatem@soton.ac.uk (A.J.T.); 2 Kinshasa School of Public Health, University of Kinshasa, Kinshasa 127, Democratic Republic of the Congo

**Keywords:** vaccination coverage, geospatial modelling, zero-dose children, under-vaccinated children, Democratic Republic of Congo, DTP vaccine, measles vaccine, dropout rate

## Abstract

**Background/Objectives:** Immunization coverage in the Democratic Republic of the Congo (DRC) consistently falls short of global standards, placing the country among those with the highest prevalence of unvaccinated or “zero-dose” children. This study aimed to generate high-resolution (1 km × 1 km) geospatial estimates of coverage for the first to third doses of diphtheria–tetanus–pertussis vaccine (DTP1–3) and the first dose of measles-containing vaccine (MCV1), along with estimates of zero-dose and under-vaccinated children to support vaccination programming in DRC. **Methods:** We used Bayesian geostatistical modelling to integrate data from the 2023 Enquête de Couverture Vaccinale (ECV) survey with geospatial covariates and harmonized population datasets. The model generated vaccine coverage and dropout rates at 1 × 1 km resolution while accounting for spatial dependence and prediction uncertainty. Grid-level estimates were aggregated to health areas, health zones, and provinces using population-weighted averages, and combined with under-one population estimates to quantify zero-dose and DTP-under-vaccinated children. **Results:** The grid-level unconstrained coverage estimates (estimation over all land grid squares) showed substantial variability across the country (DTP1: 13.3 to 99.7%; DTP2: 12.3 to 96.8%; DTP3: 3.1 to 93.6%; and MCV1: 7.5 to 93.6%). In total, 56.4% of mapped health areas achieved DTP1 coverage above 80%, but this declined to 32% for DTP2, 15% for DTP3, and 8.3% for MCV1. Around 60.5% of the health zones achieved DTP1 coverage ≥ 80%, while only 2.7% fell below 40%, indicating that substantial disparities persist across these zones. Nationally, based on constrained coverage estimates (only areas with identified built settlements) applied to an estimated 4.2 million children under 1 year in 2024, 18.4% had not received DTP1, 20.7% were DTP-under-vaccinated (received DTP1 but not DTP3), and 46.8% remained unvaccinated for MCV1. **Conclusions:** The prevalence of large numbers of zero-dose children, together with increased dropout rates, underscores systemic issues in access and follow-up initiatives. These findings are critical for microplanning and targeted outreach to achieve equitable immunization coverage in line with the Immunization Agenda 2030’s goal of leaving no child behind.

## 1. Introduction

Immunization is a vital pillar of public health, saving millions of lives and preventing countless disabilities worldwide [1,2]. The World Health Assembly in 2020 endorsed the Immunization Agenda 2030, a global strategy for 2021–2030 designed to reduce morbidity and mortality from vaccine-preventable diseases by expanding equitable access to vaccination, halving the number of unvaccinated children, and ultimately ensuring that no one is left behind [3,4,5]. This indicates that global immunization efforts are falling short of key targets, with 14.5 million zero-dose children in 2023, which is 33% more than the target, and only 84% of children receiving the third dose of DTP. Although vaccination averted 4.2 million deaths among children under 5 years of age in 2023, this is still below the nearly 4.6 million targeted, and outbreaks of vaccine-preventable diseases are rising due to stalled coverage and immunity gaps, especially in low- and middle-income countries (LMICs) [6]. Persistent gaps in access to immunization and other health services are highlighted by the fact that, as of 2024, the zero-dose and under-immunised children worldwide is 19.9 million (14.3 million zero-dose and 5.6 million partly vaccinated against the DTP vaccine), which is reduced from 21 million in 2023 (14.5 million zero-dose and 5.6 million under-vaccinated). Around 55% of these 19.9 million children reside in 10 countries: Afghanistan, Democratic Republic of the Congo (DRC), Ethiopia, India, Indonesia, Nigeria, Pakistan, the Philippines, Sudan, and Yemen [7,8].

DRC is consistently among the nations with the highest prevalence of zero-dose and under-vaccinated children [9,10,11], with roughly one in five children aged 12 to 23 months considered zero dose. Factors associated with zero-dose status include low maternal education, young maternal age, lack of civil registration, and broader system-level challenges, such as inequities in service and vaccine availability, inconsistent program management and monitoring, and issues with health worker motivation [12,13,14,15,16,17]. The prolonged absence of a national census for over 40 years, coupled with persistent conflict, has greatly exacerbated the scarcity of reliable data and complicated initiatives to vaccinate the substantial population of zero-dose children in the country [18]. Accurate and reliable immunization coverage data are essential for effective planning and resource allocation. However, historically and in general, routine immunization records in DRC have often been incomplete or unreliable, which has hindered efforts to improve vaccination coverage and contributed to a substantial number of children remaining unprotected against vaccine-preventable diseases [17,19,20]. To accelerate progress toward the Immunization Agenda 2030 goal of halving the number of zero-dose children by 2030, Gavi established the Equity Accelerator Fund (EAF), a dedicated financing mechanism designed to support countries with a high burden of zero-dose and under-vaccinated children through targeted, equity-focused interventions [21]. The EAF prioritises innovative service delivery approaches, strengthened community engagement, and focused efforts to reach populations living in remote, underserved, conflict-affected, and humanitarian settings [21].

Nationally representative household surveys are routinely implemented in many LMICs to mitigate data gaps arising from inadequate or unreliable routine reporting. However, these surveys are typically structured to be representative only at higher administrative levels, such as provinces, and fail to provide the granular data needed for planning and implementing vaccination programs at more localised levels [22,23,24]. Traditional survey data analysis methods, like direct weighted estimators, are limited to estimates at broad administrative units (e.g., provinces), often masking the fine-scale spatial heterogeneity crucial for effective immunization planning and intervention, where granular data are essential for identifying underserved populations and efficiently allocating resources [25,26,27]. These conventional approaches often fail to capture the spatial heterogeneity in immunization coverage that leads to continued disease risk, leaving clusters of zero-dose and under-vaccinated children undetected [26,28].

Recent advances in geostatistical methods, particularly Bayesian geostatistical modelling, have enhanced the ability to generate high-resolution subnational gridded estimates of vaccination coverage using geolocated cluster-level survey data and spatial covariates [26,29,30,31]. These methods create detailed maps at 1 km × 1 km resolutions, revealing that local clusters of zero-dose and under-vaccinated children often missed by traditional analyses [30]. Geospatial modelling provides a framework for producing grid-level estimates of immunization indicators, which can be aggregated to any administrative level, supporting micro-planning and targeted interventions [32,33]. Such high-resolution, spatially explicit evidence is central to the EAF approach, which emphasises data-driven identification and targeting of zero-dose and underserved populations.

Against this background, our study aimed to generate high-resolution (1 km × 1 km) estimates of DTP1–3 and MCV1 vaccination coverage across the DRC using data from the 2023 Enquête de Couverture Vaccinale (ECV) survey [34] and a Bayesian geostatistical modelling framework. Specifically, the study objectives were to: (i) estimate and map the spatial distribution of DTP 1–3 and MCV1 vaccination coverage at 1 km × 1 km resolution; (ii) assess relative dropout between successive DTP doses; and (iii) estimate the number and spatial distribution of zero-dose and under-vaccinated children at relevant administrative levels. These estimates provide immunization programme managers and policymakers with precise, location-specific information to address coverage gaps, prioritise zero-dose and under-vaccinated children, identify areas at risk of vaccine-preventable disease outbreaks, and monitor progress toward national and global immunization objectives.

## 2. Materials and Methods

**Figure 1:**
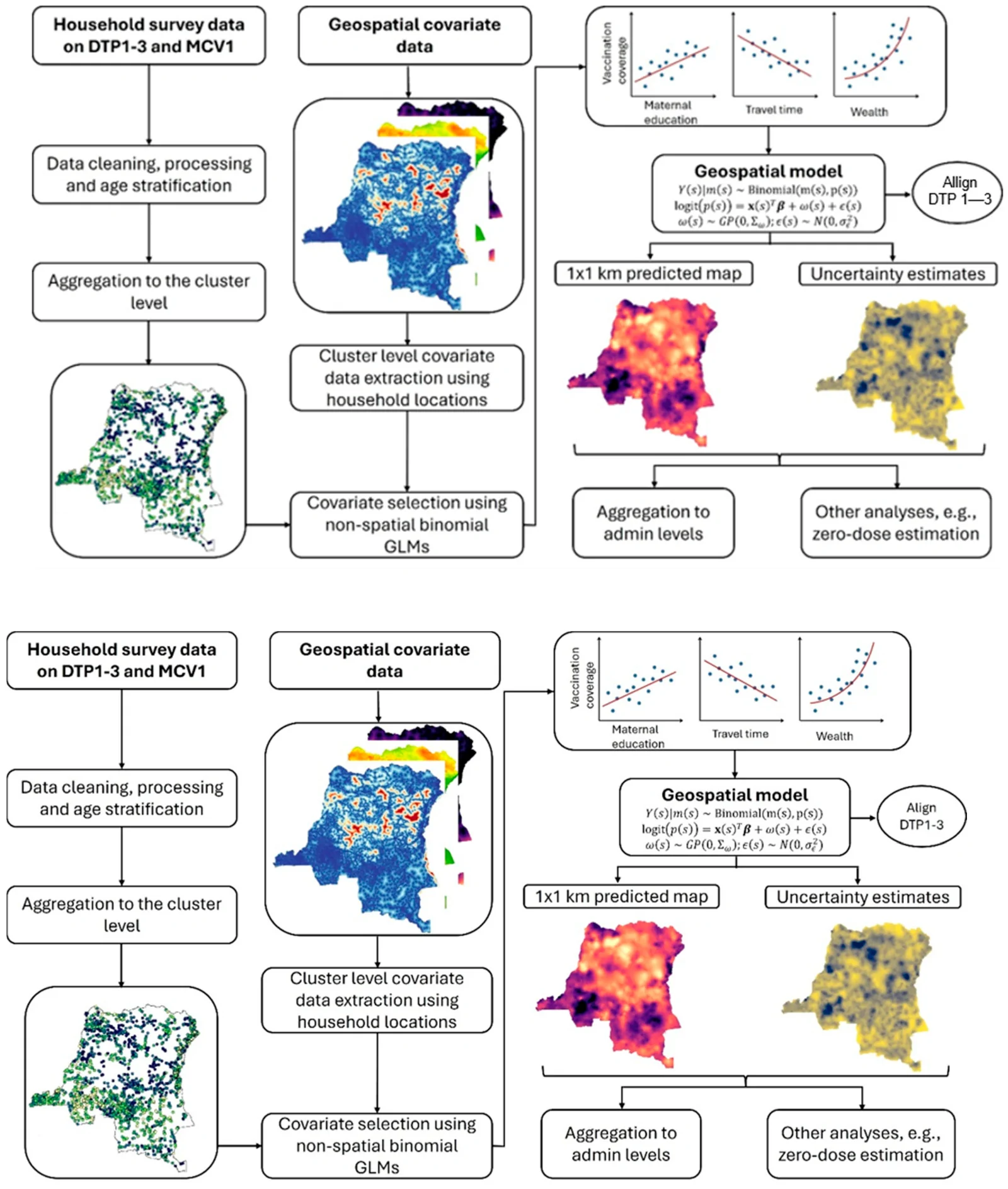
A schematic diagram of the modelling approach used to produce high-resolution estimates of vaccination coverage in DRC.

This study combined household survey data with geospatial covariates in a geospatial modelling framework to produce high-resolution estimates of childhood vaccination coverage in DRC. The workflow involved data preparation, model fitting, spatial prediction, and result interpretation, as shown in Figure 1.

### 2.1. Study Design and Data Sources

This study used data from the national immunization survey Enquête de Couverture Vaccinale (ECV), conducted by the Kinshasa School of Public Health (KSPH), referred to as ECV 2023 [34]. This survey used a multi-stage sampling method, targeting households with children aged 6–23 months in all 26 provinces of DRC (Figure S1 in the SI text), with a specific focus on children aged 12–23 months to assess complete immunization [34]. At the time of this analysis, data from the 2023–24 Demographic and Health Survey (DHS) were not yet available. Therefore, the ECV 2023 was selected as the most recent nationally representative immunization survey available for the analysis at that time.

The ECV 2023 survey used a cross-sectional, mixed-methods design that combined quantitative and qualitative aspects to evaluate routine infant vaccination coverage and identify obstacles to service utilisation. Data collection was conducted in 2023 through nationally coordinated fieldwork following standardized training, pretesting, and deployment of survey teams across all provinces [34]. The vaccination status of children was determined using both documented evidence (vaccination cards or other child health records) and maternal/caregiver recall, in accordance with the procedures specified in the ECV 2023 survey protocol [34]. Table S1 in the Supplementary Information (SI) lists the processed, age-stratified, and unweighted numbers of children sampled, along with the unweighted and weighted national-level coverage estimates from the 2023 ECV report [34]. DTP1 coverage is relatively high but drops for DTP2 and DTP3, indicating a dropout along the immunization schedule. MCV1 coverage was the lowest, with weighted national estimates for children aged 12–23 months slightly higher than unweighted values. Figure S2 in the SI displays cluster-level sample size distributions for children aged 12–23 months for the DTP1–3 and MCV1 doses. Both distributions were approximately normal, with most clusters containing 20 to 60 children. MCV1 distribution is slightly more peaked than DTP, indicating more clusters with mid-range sample sizes for MCV1. These patterns show adequate, uniform sample representation across clusters, supporting the robustness of vaccine coverage estimates.

The sampling design employed a three-stage stratified cluster sampling method, in accordance with WHO guidelines for vaccine coverage surveys [35]. Sampling clusters were defined at the health area (Aire de Santé (French term), AS) level within each Health Zone (Zone de Santé (French term), ZS). Five AS per ZS were randomly selected using the national health information system (DHIS2) as the sampling frame. Each selected AS was divided into 16 geographical segments, of which six were randomly chosen. In these segments, household enumeration identified those with at least one child aged 6–23 months. From each segment, five households were randomly selected, aiming for 30 households per health area (cluster) and 150 children per health zone, accounting for non-responses and including children aged 6–11 months. The 2023 ECV initially selected and approached 81,868 households for data collection. Following the exclusion of households without consent and those with no eligible mother/caregiver or children outside the eligible age range, the pre-cleaning dataset comprised 81,095 households, 81,937 mothers/caregivers, and 83,414 children aged 6–23 months across 2712 clusters. The data subsequently underwent extensive cleaning to address inconsistencies in individual-level records and household geolocations. Following these procedures, the final dataset comprised 79,404 households across 2656 clusters. Details of the data cleaning procedures are provided in Section S1.1 of the Supplementary Information.

The analysis focused on modelling various indicators of immunization coverage among children aged 12–23 months in DRC. These include

DTP1 coverage and unvaccinated children: DTP1 coverage is the proportion of children who received the first dose of the diphtheria–tetanus–pertussis vaccine (DTP1). The corresponding unvaccinated (zero-dose) children represent the absolute number of children under 1 year old who had not received DTP1.

DTP2 coverage and unvaccinated children: DTP2 coverage refers to the proportion of children who have received the second dose of the diphtheria–tetanus–pertussis vaccine (DTP2). The corresponding unvaccinated (DTP2 unvaccinated) children represent the absolute number of children under 1 year old who had not received DTP2.

DTP3 coverage and unvaccinated children: DTP3 coverage refers to the proportion of children who have received all three doses of the DTP vaccine. The corresponding unvaccinated children (DTP3 unvaccinated) represent the absolute number of children under 1 year old who have not received DTP3, including both zero-dose (i.e., those who did not receive the first dose) and under-vaccinated (i.e., those who received the first dose but not all three doses) children.

MCV1 coverage and unvaccinated children: MCV1 coverage refers to the proportion of children who have received the first dose of a measles-containing vaccine (MCV1). The corresponding MCV1 unvaccinated children represent the absolute number of children under 1 year old who had not received MCV1.

DTP under-vaccination and under-vaccinated children: DTP under-vaccination refers to the proportion of children who received DTP1 but did not complete the full three-dose schedule. The corresponding under-vaccinated children represent the absolute number of children under 1 year of age who received DTP1 but did not receive DTP3. In this context, under-vaccinated children correspond to the difference between DTP3 unvaccinated and DTP zero-dose children.

Relative dropout rate (proportion): Difference between the coverage of an earlier dose and that of a latter dose (earlier minus latter) of a multi-dose vaccine, divided by the coverage of the earlier dose.

### 2.2. Geospatial Covariates

To facilitate the spatial modelling of vaccination coverage, a collection of geospatial covariates, either directly associated with vaccination coverage or acting as proxies for unmeasured factors, was acquired from diverse sources, originally available at varying spatial and temporal resolutions. The geospatial covariates included indicators across six thematic categories, such as remoteness, socioeconomic status, demographics, conflict, climate, and environmental variables. All geospatial covariate data layers were processed and harmonized to a spatial resolution of 1 km × 1 km to ensure comparability across layers. Using the cleaned household GPS coordinates from the ECV 2023 survey, covariate values were extracted for each household, and a mean value was calculated for all households within each cluster, providing spatially aligned predictor variables for subsequent vaccination coverage modelling.

A total of 59 covariates were initially considered for the analysis. Covariate selection followed standard spatial modelling practices [32] using a non-spatial, frequentist framework with binomial generalized linear models (GLMs) to identify the optimal combination of predictors for modelling vaccination coverage. The geospatial covariate data were collected over different time periods between 2020 and 2024; however, most covariates had a reference year of 2023 or 2024. The relationships between individual covariates and the logit of the observed vaccination probabilities were first examined for linearity using scatter plots. Where evidence of non-linearity or significant skewness was observed, appropriate transformations (e.g., logarithmic) were applied to improve the model fit and variable distributions. To mitigate multicollinearity, pairwise correlations between covariates were calculated, and variables with strong correlations (|ρ| ≥ 0.80) were identified and excluded. The variable with the lowest Akaike Information Criterion (AIC) in the single-covariate binomial regression models was retained. Variance Inflation Factors (VIFs) were then computed as an additional diagnostic tool, and covariates with high VIF values (>4.0) were further examined. In correlated groups, the most informative covariate was selected using the AIC. A *p*-value-based backward elimination, with expert judgement, identified the most relevant covariates for modelling vaccination probability by age group. This systematic selection process resulted in 20 covariates for the spatial models (Table S2, Supplementary Information). Among the final set of 20 covariates included in the spatial models, several showed notable associations with the observed variability in vaccination coverage. Accessibility- related covariates, like walking time to health facilities, road density, and distance to urban areas, were associated with vaccination coverage. Conflict-related covariates, including distance to battles, protests, explosions, and violence against civilians, also showed notable associations with vaccination coverage.

### 2.3. Population Estimates

We used high-resolution gridded population estimates from GRID3 [36] and WorldPop Global [37], representing the 2024 population distribution, to ensure comprehensive national coverage for DRC. Bespoke population estimates were available for 15 provinces from GRID3, utilising recent operational survey data, and thus likely have better accuracy. The census projection-based WorldPop Global estimates were used for the other 11 provinces. Both datasets offer population counts at approximately 100 m spatial resolution and were harmonized by aggregating to a ~1 km grid to match the spatial resolution of the vaccination coverage predictions. Population estimates for the target age group for routine immunizations, i.e., children under one year old (U1), were derived by applying age- and sex-specific proportions from the WorldPop Global dataset to the total population grid (Figure S3 in SI text). The resulting U1 population estimates were then used as denominators for calculating the numbers of zero-dose and under-vaccinated children at the grid level. The grid-level vaccination results were finally aggregated across different administrative units, including health areas, health zones, and provinces, to facilitate programmatic use. The GRID3 dataset also contains uncertainty estimates, but neither the WorldPop Global nor the age proportions applied to the total population had estimated uncertainties. Therefore, the uncertainties for the number of children in the calculated target age-groups could not be considered in this analysis.

Ideally, the vaccination coverage estimates and the population estimates used to derive the absolute numbers of zero-dose and under-vaccinated children would be based on the same reference year. However, the most recent high-resolution population estimates available for the entire country were for 2024, whereas the vaccination coverage estimates were derived from the 2023 ECV survey. Therefore, the estimated numbers of zero-dose and under-vaccinated children represent 2024 population counts obtained by applying the 2023 modelled vaccination coverage estimates to the 2024 population. This approach assumes that demographic trends, and thus vaccination coverage, remained stable between 2023 and 2024 and did not change sufficiently over this one-year interval to materially alter the resulting estimates.

At the time of this study, health area and health zone boundaries for 14 provinces were developed and refined by GRID3 through a participatory mapping and validation process involving provincial and national health authorities, health-zone management teams, local healthcare workers, including head nurses and head doctors, and GRID3 GIS specialists. The stakeholder consultation process incorporated local knowledge of the location and extent of health facilities, settlements, administrative and service-delivery areas, and other relevant geographic features to inform the delineation of health area and health zone boundaries [38]. The resulting validated health area, health zone, and the provincial boundary datasets for the 14 provinces were provided by GRID3 and used in this study for spatial aggregation and analysis [39]. To ensure full national coverage, the remaining provincial boundaries were supplemented with OpenStreetMap (OSM)-derived data, shared via BlueSquare through GRID3. In this study, the in-depth analysis focuses on four priority provinces—Mongala, Tshopo, Maniema, and Kasaï-Oriental—identified by Gavi as priority areas due to relatively low vaccination coverage and higher dropout rates. The selection of these provinces allows us to illustrate the spatial modelling approach and highlight areas where programmatic interventions are most needed. Analyses can be extended to other provinces using the same methods, and the insights from these priority provinces are broadly applicable across DRC.

### 2.4. Model Fitting and Validation

We fitted Bayesian binomial geostatistical models to estimate the coverage of DTP1–3 and MCV1 across DRC. For cluster location $s_{i},\;i=1,\ldots ,m$, let $Y(s_{i})$ denote the number of children vaccinated out of $n(s_{i})$ sampled. The model assumes that $Y\left(s_{i}\right)|p\left(s_{i}\right)∼\mathrm{Binomial}\left(n\left(s_{i}\right),p\left(s_{i}\right)\right)$, where $p\left(s_{i}\right)$ represents the true vaccination coverage at the location $s_{i}$. A logistic regression model was then specified for $p\left(s_{i}\right)$ such that

(1) $$\mathrm{logit}\left(p\left(s_{i}\right)\right)=x{\left(s_{i}\right)}^{\prime }\beta +\omega \left(s_{i}\right)+\epsilon \left(s_{i}\right)$$

$x\left(s_{i}\right)$ is a vector of covariates with associated regression coefficients β, $\epsilon \left(s_{i}\right)$ is an independent and identically distributed Gaussian random effect with variance $\sigma_{\epsilon }^{2}$, and $\omega \left(s_{i}\right)$ is a spatially structured Gaussian random effect. The spatial effect was assumed to follow a zero-mean Gaussian process with Matérn covariance function:

(2) $$\Sigma_{\omega }\left(s_{i},s_{j}\right)=\sigma^{2}\frac{1}{2^{\nu -1}\Gamma \left(\nu \right)}{\left(\kappa ||s_{i}-s_{j}||\right)}^{\nu }K_{\nu }\left(\kappa ||s_{i}-s_{k}||\right)$$

where $\left||s_{i}-s_{j}|\right|$, denotes the Euclidean distance between cluster locations $s_{i}$ and $s_{j}$. $\sigma^{2}$ is the marginal variance, κ is the scaling parameter related to the spatial range *r* ($r=\frac{\sqrt{8\nu }}{\kappa }$) and $K_{\nu }$ is the modified Bessel function of the second kind. For identifiability, we fixed ν = 1. The models were implemented using the integrated nested Laplace approximation (INLA) with the stochastic partial differential equations (SPDE) approach in the R-INLA package (R-INLA Project), which provides computationally efficient inferences for latent Gaussian models.

We followed the ratio-based approach [40] when modelling DTP1–3 to ensure that the estimates of these indicators were internally consistent, i.e., $p_{1}\left(s\right)\geq p_{2}\left(s\right)\geq p_{3}\left(s\right)$, where $p_{1}\left(s\right)$, $p_{2}\left(s\right)$ and $p_{3}\left(s\right)$ denote the respective coverages of the three-dose DTP vaccination series at the spatial location s. With the ratio-based approach, two ratio indicators, namely the ratio of DTP2 to DTP1 (DTP2/DTP1) and the ratio of DTP3 to DTP2 (DTP3/DTP2), are directly modelled alongside DTP1, from which the estimates of DTP2 and DTP3 are derived [40].

We assessed the predictive performance of the fitted models using a 10-fold cross-validation procedure at the cluster level. The n cluster locations were randomly partitioned into 10 approximately equal subsets. For each fold, the model was fitted to the data from all but one subset and validated on the remaining subset, with the process repeated until all clusters were used for validation.

Let $p\left(s_{i}\right)$ denote the observed vaccination coverage in the cluster $s_{i}$ and $\hat{p}\left(s_{i}\right)$ the corresponding model prediction. For each cross-validation fold, we computed the following evaluation metrics:

The average bias (AvBias) measures the mean difference between predicted and observed coverage, indicating whether the model systematically overestimates or underestimates vaccination coverage:

(3) $$\mathrm{AvBias}=\frac{1}{m_{c}}\sum_{i=1}^{m_{c}}\left(\hat{p}\left(s_{i}\right)-p\left(s_{i}\right)\right)$$

The root mean squared error (RMSE) quantifies the overall magnitude of prediction errors, giving greater weight to larger discrepancies:

(4) $$\mathrm{RMSE}=\sqrt{\frac{1}{m_{c}}\sum_{i=1}^{m_{c}}{\left(\hat{p}\left(s_{i}\right)-p\left(s_{i}\right)\right)}^{2}}$$

The mean absolute error (MAE) measures the average absolute deviation between predicted and observed coverage:

(5) $$MAE\;=\frac{1}{m_{c}}\;\sum_{i=1}^{m_{c}}\left|\hat{p}\left(s_{i}\right)-p\left(s_{i}\right)\right|$$

We also calculated the Pearson correlation coefficient between observed and predicted values to assess the strength of linear association. Here, $m_{c}$ denotes the number of validation locations in each fold.

All four metrics evaluate point prediction accuracy: smaller AvBias (in absolute terms), RMSE, and MAE values indicate better performance, while larger correlations indicate stronger agreement between observed and predicted coverage. Results were averaged across cross-validation folds for overall performance. At the provincial level, modelled estimates of vaccination indicators were compared with direct survey estimates to assess external validity.

## 3. Results

### 3.1. Model Validation

To evaluate the performance and reliability of the geostatistical model, we conducted validation at various levels. This included cluster-level cross-validation, provincial-level comparisons with survey estimates, and national-level comparisons, ensuring the model accurately reflects immunization patterns across the DRC.

#### 3.1.1. Cluster-Level Cross-Validation

Model performance was first evaluated at the cluster level using k-fold cross-validation for key immunization indicators, including DTP1, MCV1, and the ratio-indicators DTP2/1 and DTP3/2 (Table 1).

Among all the indicators evaluated, DTP1 showed the strongest model performance, with a high correlation of 0.760, low RMSE (0.146) and MAE (0.108), and a negligible average bias of 0.004. These results demonstrate high predictive accuracy and minimal systematic deviation between predicted and observed values. For MCV1, concordance with the observed data was moderate with a correlation of 0.657. However, its RMSE (0.195) and MAE (0.156) were the highest among the indicators, suggesting a comparatively greater uncertainty in the model’s predictions for this indicator. Despite this, the average bias for MCV1 remained minimal at 0.002, indicating that predictions were not systematically biased in any direction. The ratio indicators, DTP2/1 and DTP3/2, exhibited lower correlations of 0.531 and 0.496, respectively. These numbers indicate greater difficulty in predicting these indicators than with first-dose coverage. Consequently, both RMSE and MAE values were elevated for these indicators, while average biases remained very low, almost zero, indicating models did not consistently overestimate or underestimate coverage.

In-sample prediction plots for DTP1 and MCV1 (Figure S4 in SI text) showed generally good agreement between observed and predicted coverage, with DTP1 displaying closer correspondence, as points clustered more tightly along the diagonal compared to MCV1. These results were consistent with the out-of-sample predictions obtained through k-fold cross-validation, with DTP1 showing the highest accuracy among all indicators (Table 1). In general, the model demonstrated good predictive accuracy across all modelled indicators, although predictive performance was lower for the ratio indicators (DTP2/1 and DTP3/2) than for DTP1.

#### 3.1.2. Provincial Level Validation

**Figure 2:**
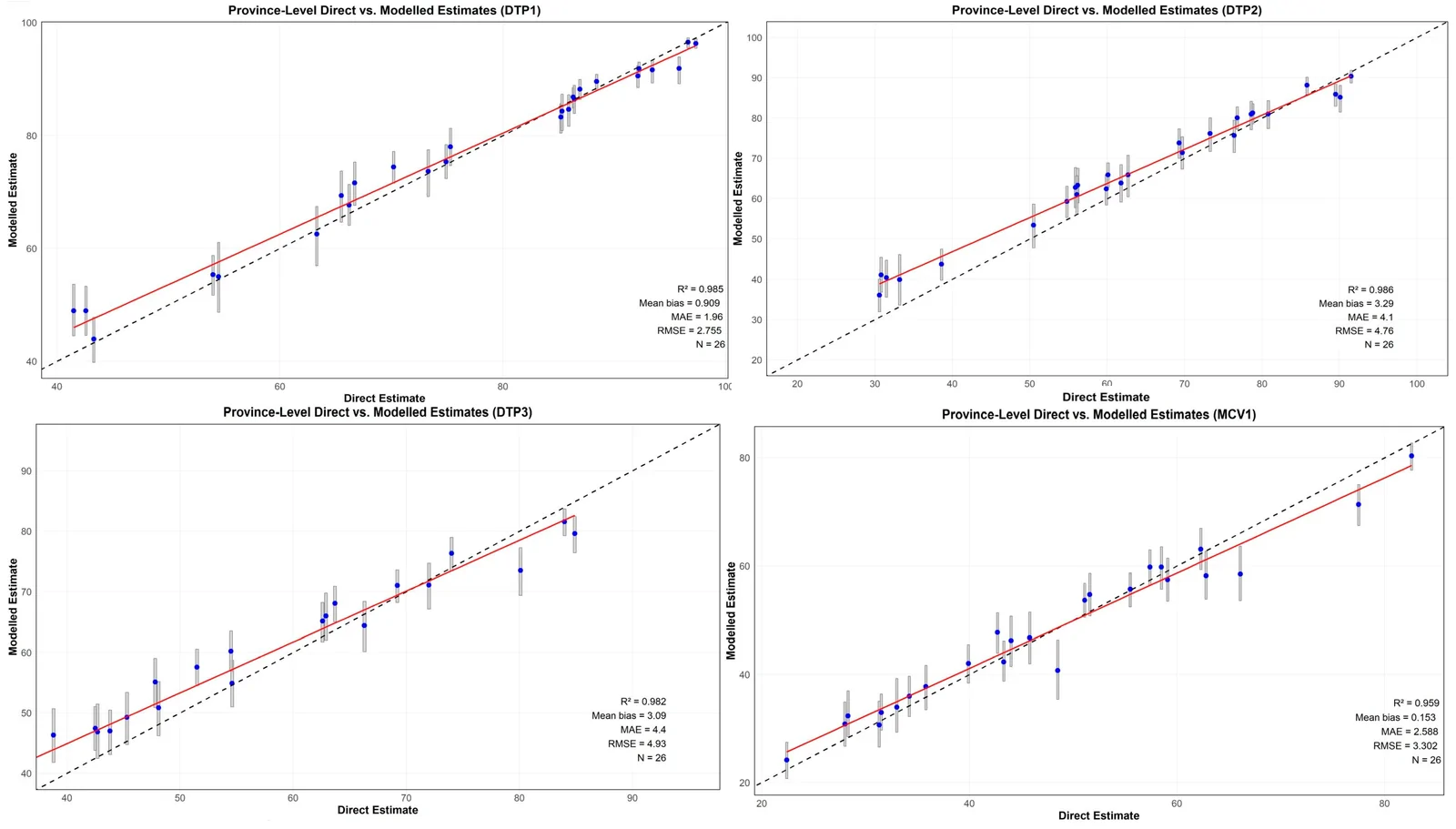
Model validation (modelled vs. direct survey estimates) at the provincial level for DTP1, DTP2, DTP3, and MCV1.

To further assess the validity and accuracy of the spatial model used to estimate vaccine coverage, we compared modelled provincial-level vaccination coverage estimates (%) with direct survey-based estimates (%) for DTP1–3 and MCV1 vaccines (Figure 2). Each blue point represents a province in DRC, with vertical lines indicating the 95% credible intervals around the modelled estimates. The *x*-axis shows the direct survey-based vaccination coverage (%), while the *y*-axis shows the corresponding modelled vaccination coverage (%). The black dashed line represents the line of equality (*y* = *x*), indicating perfect agreement, while the red line represents the fitted linear regression between direct and modelled coverage estimates.

For DTP1 (top-left panel), modelled estimates match direct survey estimates across the coverage range (R^2^ = 0.985), showing strong linearity and minimal deviation. This suggests the model accurately captures coverage for the first dose of the DTP vaccine. DTP2 (top-right panel) also showed strong agreement (R^2^ = 0.986), though some provinces show minor deviations, especially at low coverage (30–45%). DTP3 (bottom-left panel) showed similarly strong agreement (R^2^ = 0.982), but the data showed greater dispersion, with modelled estimates often exceeding survey values at intermediate levels. MCV1 (bottom-right panel) demonstrated strong agreement (R^2^ = 0.959), but some provinces diverge significantly at mid-coverage (40–60%). These comparisons demonstrate that the geostatistical model consistently replicates provincial-level coverage estimates from survey data.

#### 3.1.3. National-Level Validation

Table 2 shows national-level direct estimates from the 2023 ECV report [34] and national-level modelled estimates for DTP1–3 and MCV1. At the national scale, the modelled vaccination coverage estimates closely match those from the ECV report, confirming the validity of the geostatistical model in capturing overall immunization patterns across DRC.

The differences between modelled and direct national estimates were minimal, ranging from 0.5 to 2.8 percentage points across the four vaccines. This strong concordance highlights the credibility of the geostatistical model in replicating direct survey results at a macro scale and its applicability for generating high-resolution, subnational coverage estimates where direct data may be sparse or inaccurate.

### 3.2. High-Resolution Coverage Estimates

**Figure 3:**
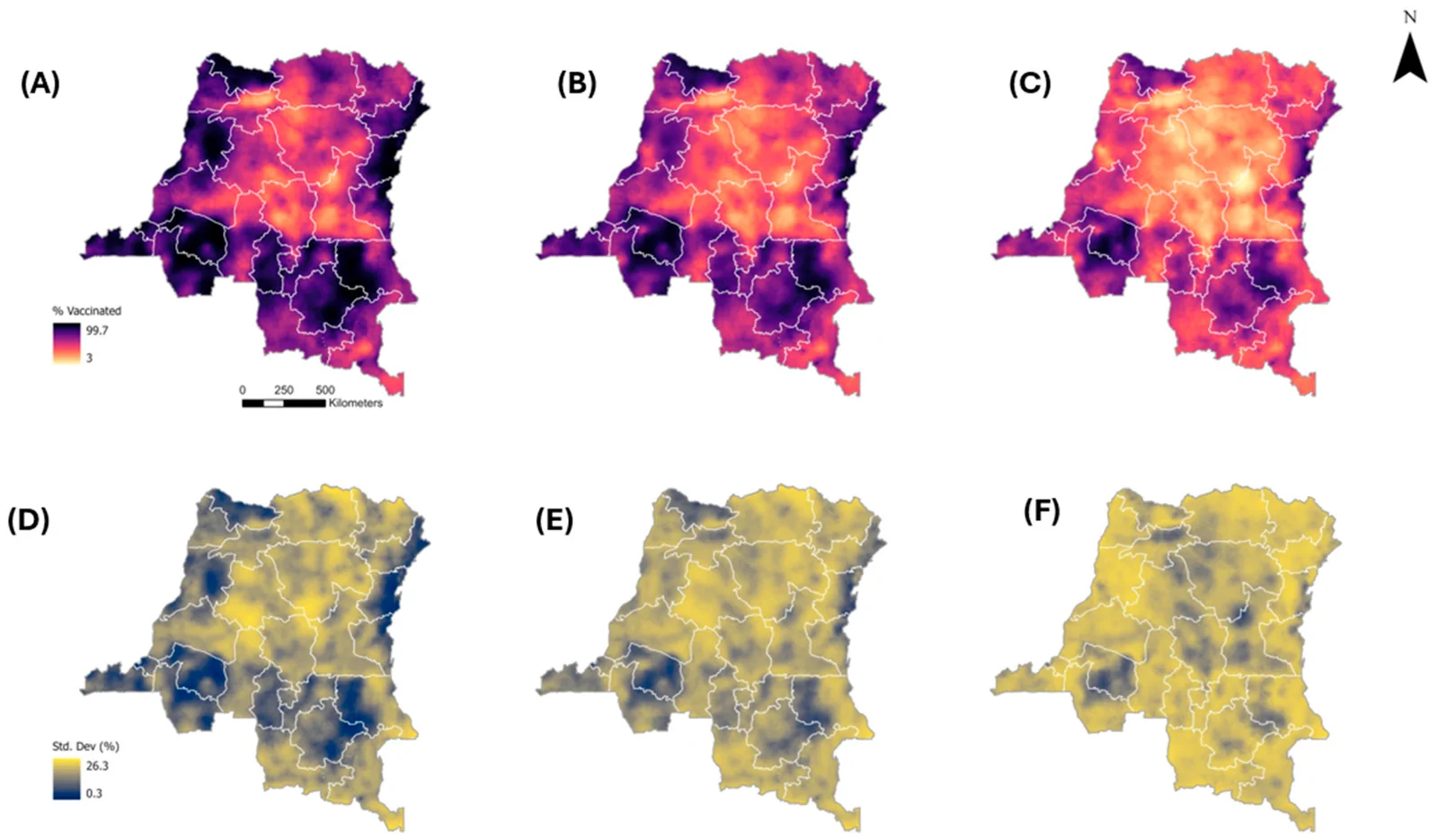
Estimated DTP1–3 coverage and associated uncertainties are shown as standard deviations at 1 km × 1 km resolution for DRC in 2023. Panels A–C show the estimated percentage of children aged 12–23 months who received DTP1 (**A**), DTP2 (**B**), and DTP3 (**C**). For Panels A–C, darker shading indicates higher estimated vaccination coverage, whereas lighter shading indicates lower coverage. Panels D–F show the corresponding uncertainties in coverage for DTP1 (**D**), DTP2 (**E**), and DTP3 (**F**), expressed as standard deviations (%). For Panels D–F, lighter shading (yellow) indicates higher uncertainty (larger standard deviations), whereas darker shading (blue) indicates lower uncertainty.

**Figure 4:**
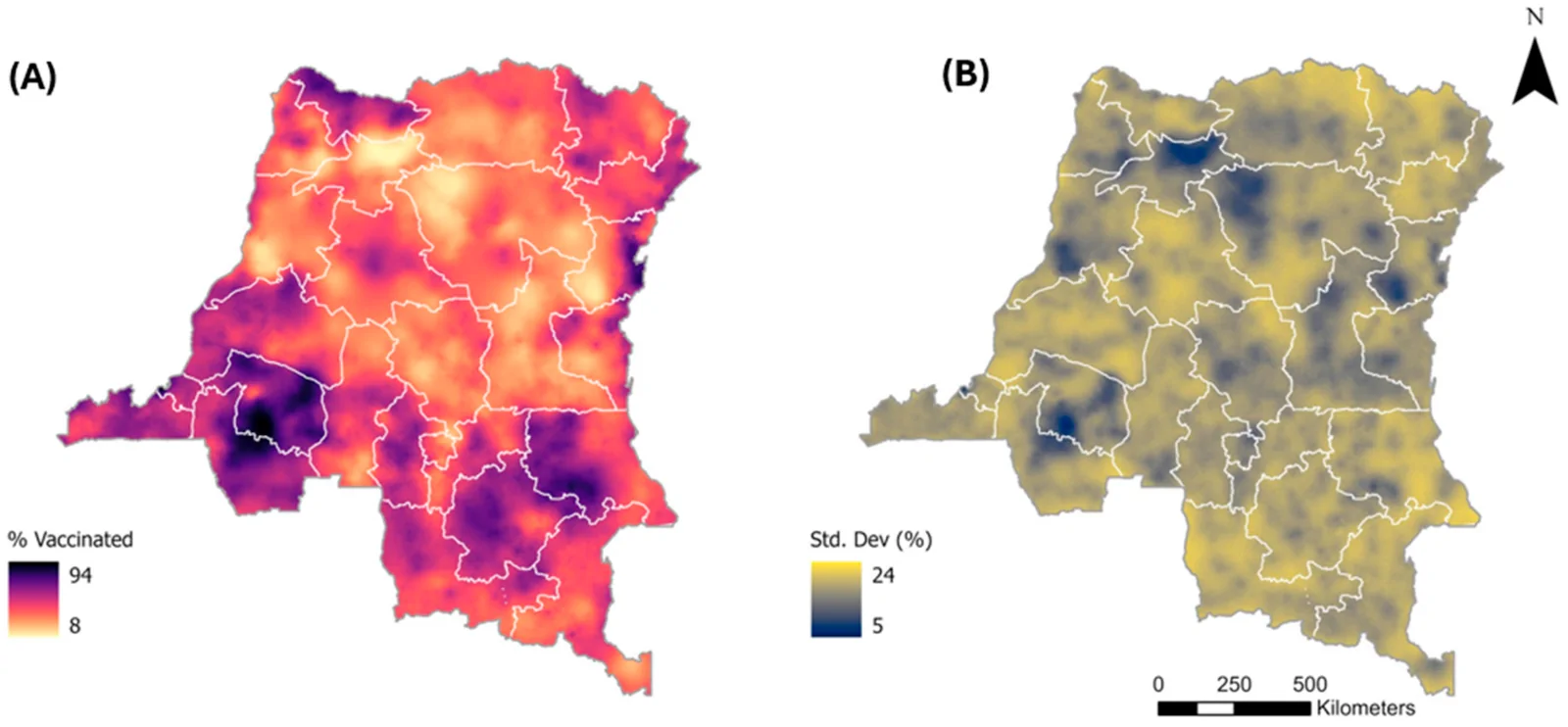
Estimated MCV1 coverage and associated uncertainties shown as standard deviations at 1 km × 1 km resolution for DRC in 2023. Panel (**A**) shows the estimated percentage of children aged 12–23 months who received the first dose of measles-containing vaccine (MCV1). For Panel (**A**), darker shading indicates higher estimated vaccination coverage, whereas lighter shading indicates lower estimated coverage. Panel (**B**) shows the corresponding uncertainty in MCV1 coverage, expressed as the standard deviation (%). For Panel (**B**), lighter shading (yellow) indicates higher uncertainty (larger standard deviations), whereas darker shading (blue) indicates lower uncertainty (smaller standard deviations).

**Figure 5:**
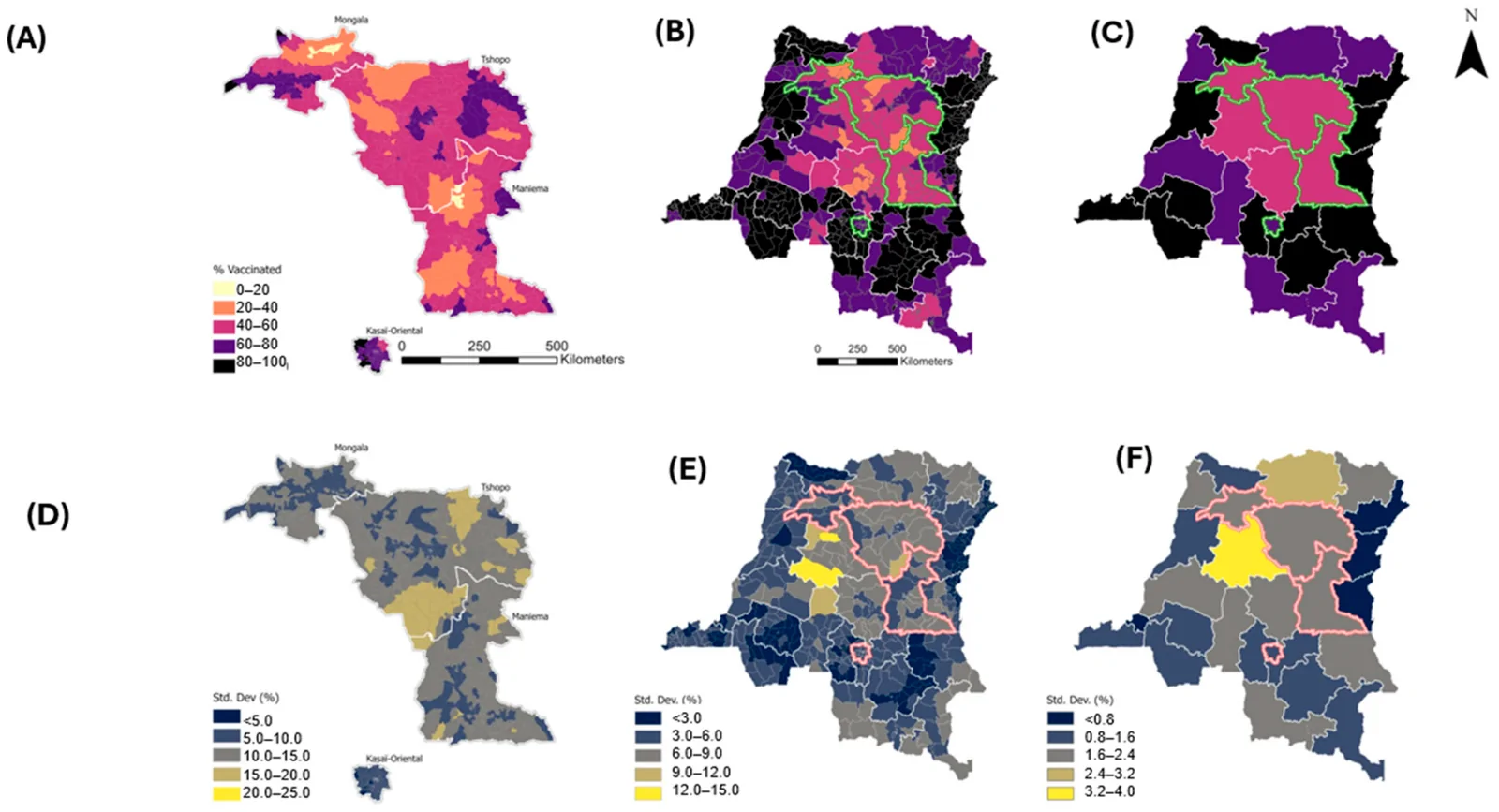
Estimated DTP1 coverage and associated uncertainties among children aged 12–23 months in DRC in 2023. Panels (**A**–**C**) show estimated DTP1 vaccination coverage (%) at the health area (**A**), health zone (**B**), and provincial (**C**) levels. Health area estimates (**A**,**D**) are shown for four priority provinces (Mongala, Tshopo, Maniema, and Kasaï-Oriental), while health zone and provincial estimates (**B**,**C**,**E**,**F**) are presented at the national level. Panels (**D**–**F**) show the corresponding uncertainty in DTP1 coverage, expressed as the standard deviation (%), at the health area (**D**), health zone (**E**), and provincial (**F**) levels.

High resolution (1 km × 1 km) gridded maps of DTP1–3 and MCV1 coverage were generated for the entire DRC (Figure 3 and Figure 4), with results aggregated to health area, health zone, and provincial levels (Figure 5). The model first generated unconstrained vaccination estimates, predicting coverage across all land grid cells within administrative boundaries, regardless of the presence of mapped human settlements. These unconstrained estimates (Figure 3 and Figure 4) provide a complete representation of coverage patterns and associated uncertainty. To produce population-relevant summaries, unconstrained coverage surfaces were combined with settlement-constrained population estimates, restricting estimates to grid cells with built settlements. These constrained estimates were then aggregated to health area, health zone, and provincial levels (Figure 5) and used to calculate population-weighted coverage levels. While unconstrained estimates characterise continuous spatial patterns and geographic heterogeneity, constrained estimates provide more realistic population-weighted results and were used to calculate national and subnational vaccination coverage levels and the numbers of vaccinated, under-vaccinated, and zero-dose children reported in this study. Figure 3 shows unconstrained estimates of DTP1–3 coverage and associated uncertainties (expressed as standard deviations) at a 1 km × 1 km resolution. Darker shades indicate higher coverage, while uncertainty maps highlight areas with more uncertain predictions.

Spatially unconstrained coverage estimates at the grid level (1 km × 1 km) demonstrated significant variability, with DTP1 coverage ranging from 13.3% to 99.7%, DTP2 from 12.3% to 96.8%, and DTP3 from 3.1% to 93.6%. The lowest DTP1 coverage was observed in the provinces of Mongala, Tshopo, Maniema, Tshuapa, and Sankuru. For all three DTP doses, the highest coverage was noted in certain urbanized and well-connected areas, especially in the central-western and southern provinces, where some grid cells had modeled coverage estimates of 95% or more. In contrast, extensive areas in the more remote northern and eastern provinces exhibited significantly lower coverage, sometimes falling below 15% for the later doses (DTP2 and DTP3). As we progress to DTP3, coverage appears to decline in some regions, suggesting gaps in vaccination. The areas with the lowest DTP3 coverage are predominantly in the central-northern and northeastern regions, encompassing large parts of the provinces of Mongala, Tshopo, Bas-Uélé, and Haut-Uélé. Additional pockets of low coverage are evident across the central forest belt, particularly in Tshuapa and Sankuru, extending into parts of Maniema. The uncertainty map reveals high uncertainty across vast portions of the northern, western, and southeastern regions. In the far northeast, provinces such as Ituri, Haut-Uele, Bas-Uele, and Nord Kivu exhibit higher standard deviations. Similarly, broad areas of elevated uncertainty were observed in the southeastern provinces of Kasai-Oriental, Kasai-Central, Lomami, Lualaba, and the western provinces of Mai-Ndombe and Equateur.

Figure 4 shows the estimated MCV1 unconstrained coverage and its associated uncertainties (expressed as standard deviations) at a 1 km × 1 km resolution. For MCV1 coverage, estimates range from 7.5% to 93.6%, with the highest levels concentrated in parts of the south and west. However, it is important to note that areas of very low coverage tend to be sparsely populated, thus contributing less to national-level estimates. This pattern is further clarified by the settlement-constrained MCV1 coverage estimates, which adjust spatial estimates based on population distribution and are provided for four provinces in the Supplementary Material (Figure S5 in SI text). These maps show that regions with low modelled vaccination coverage often correspond to areas with low population density. The uncertainty pattern for MCV1 coverage was similar to that for DTP coverage, with higher standard deviations in peripheral and low-access regions, highlighting geographic disparities in both vaccination coverage and the robustness of estimates.

Following the generation of 1 km × 1 km grid-level immunization coverage predictions for DTP1, DTP2, DTP3, and MCV1, all estimates were aggregated to health areas, health zones, and provinces using population-weighted averages of grid-level coverage. Importantly, constrained population layers were used as weights in all aggregation steps, such that grid cells with zero or negligible population contributed little or nothing to the resulting administrative-level coverage estimates. As a result, all reported coverage percentages and threshold-based summaries reflect vaccination performance among populated areas. Figure 5 shows the estimated DTP1 coverage and associated uncertainties (expressed as standard deviations) among children aged 12–23 months at these three administrative levels in DRC. Health area-level estimates are presented for four priority provinces (Mongala, Tshopo, Maniema, and Kasaï-Oriental) to enable clearer visualization at fine spatial resolution, whereas health zone and provincial estimates are shown for the entire country. For DTP1, over half (56.4%) of health areas reported 80–100% coverage, while this proportion declined to 32% for DTP2, 15% for DTP3, and 8.3% for MCV1 (for DTP2–3 and MCV1, see Table S3 and Figures S6–S8 in SI text).

At the health area level within the four study provinces (n = 1330), the population -weighted constrained estimates revealed substantial local heterogeneity in DTP1 coverage. Nearly three-fifths (58.5%) of health areas had DTP1 coverage below 60% (based on population-weighted, constrained estimates), including 17.0% with coverage between 20% and 40% and 41.5% between 40% and 60%. Only 7.4% of health areas achieved the ≥80% threshold, highlighting pronounced localized gaps in vaccine delivery and access. In contrast, at the national level, patterns differ when examined at coarser administrative scales. At the health zone level nationally (n = 519), most zones (60.5%) achieved high DTP1 coverage (≥80%), while only 2.7% fell below 40%. This contrasts with the health area results, suggesting that although many zones meet administrative targets, substantial within-zone disparities persist, reflecting uneven performance among constituent health areas. At the provincial level nationally (n = 26), the spatial gradient becomes more generalized: 50% of provinces achieved ≥80% coverage, 30.8% reported 60–80%, and 19.2% remained in the 40–60% range. Higher-performing provinces were concentrated in the southeastern part of the country, whereas several central and western provinces exhibited consistently moderate or low coverage levels.

The uncertainty maps indicate that prediction uncertainty diminishes with larger administrative units. At the health area level (four provinces), standard deviations exceeding 15% are noted in certain remote or sparsely populated regions. Nationally, at the health zone level, most zones exhibit uncertainty below 9%, while at the provincial level, uncertainty remains consistently low, typically under 4%, reflecting increased confidence in estimates at aggregated scales. These findings illustrate that although vaccination coverage appears satisfactory at higher administrative levels, significant heterogeneity and low coverage persist at the local (health area) level, underscoring the importance of conducting analyses at fine spatial scales to effectively target immunization efforts.

Figures S6 and S7, along with Table S3 in the SI text, present the aggregated results for DTP2 and DTP3. At the national level, aggregation reduced fine-scale heterogeneity, but substantial geographic disparities persisted, with DTP2 coverage consistently higher than DTP3 across health areas, health zones, and provinces. Coverage was generally higher in southeastern DRC, whereas central and western regions continued to show lower coverage, highlighting persistent inequalities and gaps in completion of the DTP series. Figure S8 in the SI text shows the estimated MCV1 coverage, along with the associated uncertainties (standard deviations), at the health area, health zone, and province levels in the DRC. At the national level, MCV1 coverage was particularly low at finer spatial scales: 85.8% of health areas reported coverage below 40%, while only 6.9% of health zones reached 80% or more. Higher MCV1 coverage was mainly concentrated in southern and western DRC, whereas central and northern regions (Sud Ubangi, Équateur, Mongala, Bas-Uélé, Tshopo, Sankuru, and Maniema) showed persistent gaps; uncertainty was generally low, with most health zones having standard deviations below 6% and most provinces below 2.4%.

### 3.3. Relative Dropout Rates Between DTP Doses

**Figure 6:**
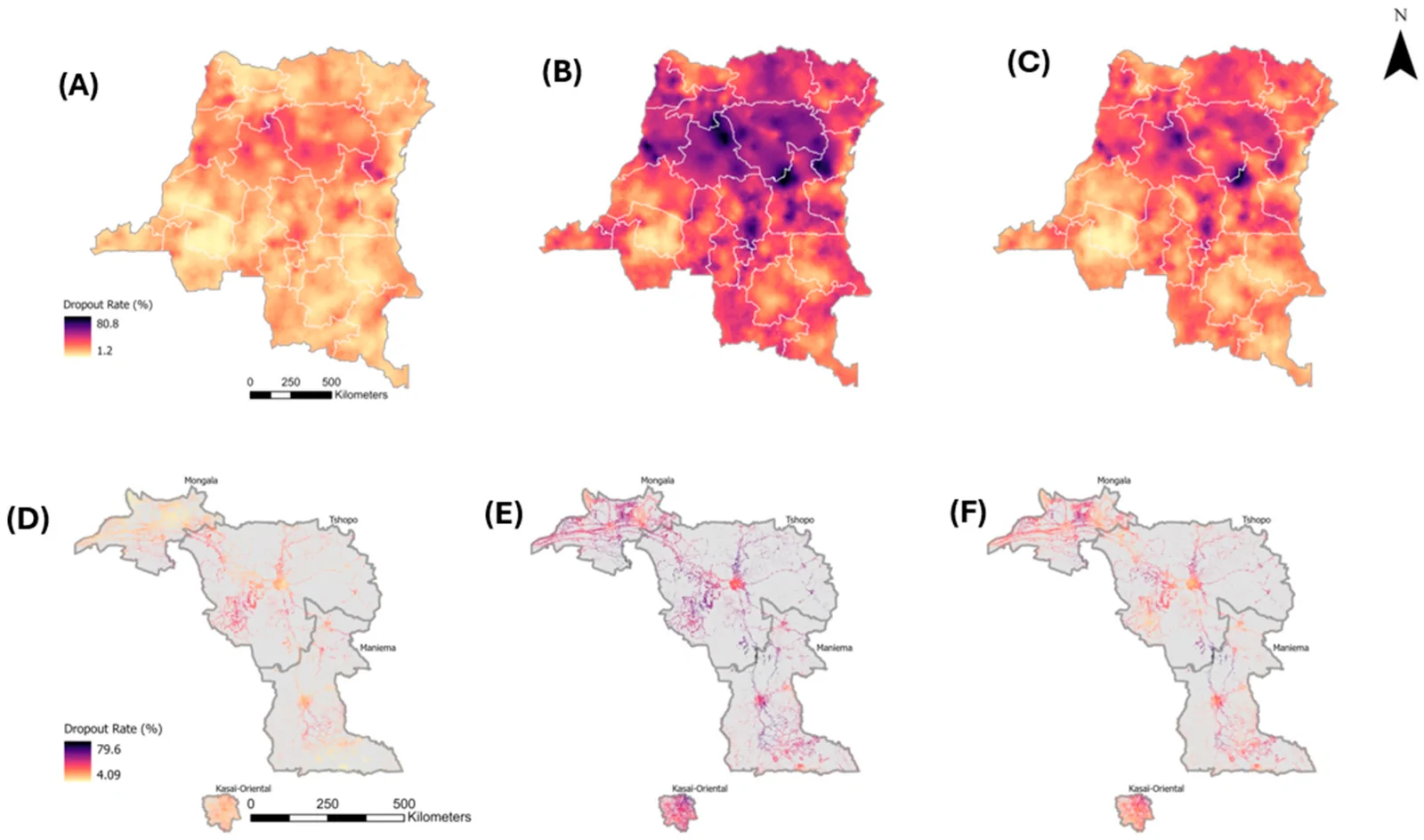
Estimated (national) and settlement-constrained (priority provinces) relative dropout rates between DTP doses for DRC in 2023. Panels (**A**–**C**) show national relative dropout rates between DTP1–DTP2 (**A**), DTP1–DTP3 (**B**), and DTP2–DTP3 (**C**). Panels (**D**–**F**) show settlement-constrained relative dropout rates within priority provinces between DTP1–DTP2 (**D**), DTP2–DTP3 (**E**), and DTP1–DTP3 (**F**).

Dropout rates are critical for assessing immunization programmes because they identify not only where children are missing their first dose, but also where follow-up systems are failing to retain them through subsequent contacts. DTP dropout maps in Figure 6 quantify differences in the proportions of children who did not complete the three-dose series, dropping out after either the first or the second dose.

The top panels (Figure 6) show the estimated relative dropout rates between DTP1–DTP2 (left), DTP2–DTP3 (centre), and DTP1–DTP3 (right) across DRC at a 1 km × 1 km spatial resolution. Across all three maps, dropout values range from approximately 3% to over 80%, with lighter yellow shades indicating low dropout and darker purple shades indicating high dropout. The map for dropout between the first and second doses shows that much of the country experiences relatively low to moderate attrition early in the vaccination schedule. In many regions, children who receive their first dose are likely to continue to the second dose, although localized areas of weak early follow-up remain evident. The pattern changes markedly in the map showing dropout between the second and third doses. Darker, more extensive areas were observed, indicating a higher proportion of children failing to complete the full vaccination series. Greater dropout rates were found between DTP2 and DTP3 than between DTP1 and DTP2, particularly in the northern provinces. The dropout rates between DTP1 and 3 are highest in areas of lower coverage in the provinces of Mongala, Tshuapa, Tshopo, Sankuru, and Maniema.

The bottom panels in Figure 6 show the settlement-constrained dropout rate maps for the same dose comparisons, focusing on four provinces: Mongala, Tshopo, Maniema, and Kasaï-Oriental. Dropout rates ranged from 4% to 79.6%, with lighter yellow areas representing lower dropout and darker purple areas indicating higher dropout. Distinct clusters of elevated dropouts were visible in the central Mongala, northwestern Tshopo, and parts of southern Maniema, especially in low-density, hard-to-reach areas. The constrained maps emphasize that many of these high-dropout areas coincide with sparsely populated and remote locations, which are often characterised by multiple factors such as service availability, socio-economic conditions, geographic accessibility and other barriers that may hinder immunization follow-up. By distinguishing between early- and late-series dropout, these maps provide valuable insights into whether the main barrier is initial access to services or the ability to maintain engagement over time.

### 3.4. Estimates of Numbers of Zero-Dose and Under-Vaccinated Children

**Figure 7:**
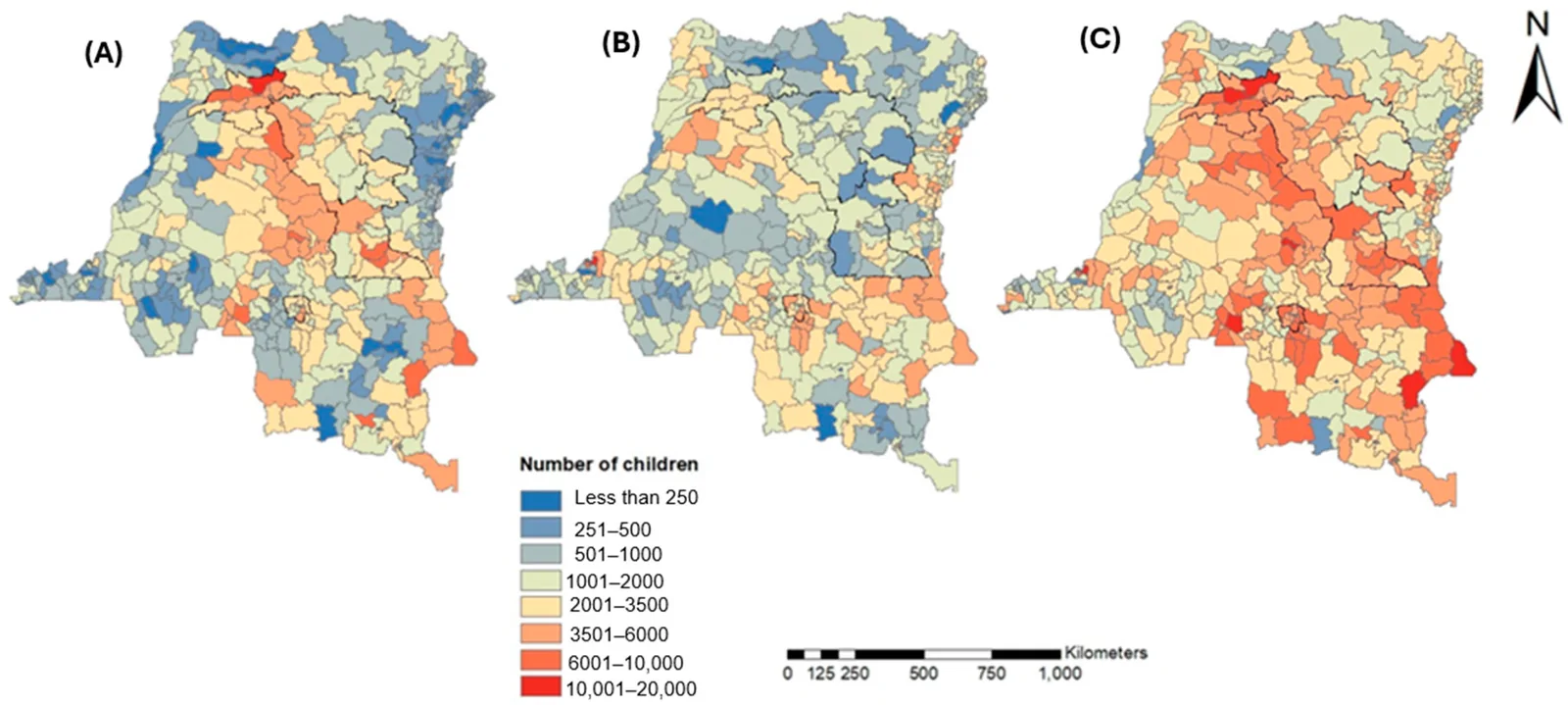
Estimated spatial distribution of DTP vaccination gaps among children aged <1 year by health zone in DRC, 2024. Panels show (**A**) estimated DTP zero-dose children, (**B**) estimated DTP under-vaccinated children, and (**C**) estimated DTP3-unvaccinated children.

To quantify the burden of zero-dose children—those who had not received any doses of DTP—we combined the modelled vaccination coverage estimates with high-resolution gridded population data for children under one year of age (Figure S2 in SI text). This analysis was conducted at the 1 km × 1 km grid-cell level and subsequently aggregated to various administrative levels for reporting. Because vaccination coverage was derived from the 2023 ECV survey of children aged 12–23 months, whereas the population denominator represents children under one year in 2024, the resulting numbers should be interpreted as estimated numbers of children under one year without the respective vaccination, rather than direct counts of unvaccinated children in 2024. The resulting estimates of DTP unvaccinated, DTP under-vaccinated, and DTP3 unvaccinated children under one year of age by health zone are shown in Figure 7. The estimated numbers should be interpreted as best estimates rather than exact counts. While uncertainty was quantified for the modelled vaccination coverage estimates, we were unable to fully quantify uncertainty around the estimated numbers of unvaccinated/under-vaccinated children. This is because uncertainty estimates were only available for the total population estimates, not for the under-1 population estimates. The under-1 population estimates were derived by applying age–sex proportions to the total population estimates, and as a result, uncertainty estimates were not generated for them. This limitation should therefore be considered when interpreting the estimated numbers and when using these estimates to inform programme planning and resource allocation.

At the national level, applying the modelled coverage estimates to the 2024 population of children under one year (total U1 population: 4,209,457) resulted in an estimated 775,023 children (18.4%) without DTP1 (DTP unvaccinated/zero-dose) and 1,969,969 children (46.8%) without MCV1. Furthermore, an estimated 870,537 children (20.7%) were DTP under-vaccinated, having received DTP1 but not DTP3. A total of 1,645,560 children (39.1%) were estimated to be DTP3 unvaccinated, having not received DTP3.

The highest estimated numbers of DTP unvaccinated (zero-dose) children are concentrated in specific health zones, with the largest burden in Yambuku (Mongala), where an estimated 11,871 children are zero-dose. Other health zones with very high numbers include Lodja in Sankuru, Kindu in Maniema, Lisala in Mongala, Kamwesha in Kasai, Kilwa in Haut-Katanga, and Kunda in Maniema, with counts ranging from 6501 to 11,871. At the provincial level, the highest estimated numbers of DTP unvaccinated (zero-dose) children (>50,000 under 1 s) were estimated in Mongala, Tshopo, Maniema, Sankuru, Kasaï, and Haut-Katanga provinces. Our results suggest that these areas represent priority provinces for outreach and initial contact with vaccination services, as the model estimates a high burden of children under one year who had not received DTP1. As an illustration of how these estimates can inform spatial prioritization within provinces, Figure S9 in the SI text highlights priority health zones in Maniema. Combining the spatial map with the population-adjusted bar chart helps identify priority health zones where targeted outreach could have the greatest impact on reducing the zero-dose burden. Although Maniema is presented as an example, this approach can be applied across all provinces to support data-driven microplanning.

The estimated number of DTP under-vaccinated children, defined as those who received DTP1 but did not receive DTP3, was generally lower than the estimated number of DTP unvaccinated (zero-dose) children. At the health zone level, several regions show high concentrations of under-vaccinated children, indicating gaps in completion of the vaccination schedule. Nsele (Kinshasa) has the highest number of under-vaccinated children (16,822), followed by Mutwanga (Nord Kivu) with 6429 and Ngafula (Kinshasa) with 6200. These numbers indicate systemic obstacles in ensuring follow-up and adherence to the immunization schedule, even where initial access is achieved.

The estimated number of DTP3 unvaccinated children, defined as children who had not received DTP3 and therefore including both DTP unvaccinated (zero-dose) and DTP under-vaccinated children, indicates the extent of children without full protection against DTP-related diseases. Nsele health zone in Kinshasa has the highest count with 19,196 un- and under-vaccinated children, representing 25.0% of the under-1 population. Several other zones also show both high counts and high percentages of unvaccinated children, indicating significant coverage gaps relative to their population sizes. Other health zones with particularly high estimated numbers included Kilwa in Haut-Katanga, with 14,108 DTP3 unvaccinated children (61.6% of U1), and Kindu in Maniema with 14,090 (64.6% of U1). Yambuku in Mongala had the highest proportion of DTP3-unvaccinated children, with 85.8% of the under-1 population estimated to be unvaccinated, followed by Djolu in Tshuapa (84.9%) and Lodja in Sankuru (74.2%). These findings show that several health zones especially in Mongala, Tshuapa, Sankuru, and Kasaï, continue to experience critical gaps in vaccine coverage, emphasizing the need for targeted immunization interventions.

## 4. Discussion

This study provides high-resolution estimates of DTP1–3 and MCV1 coverage across DRC, offering insights into spatial heterogeneity in immunization coverage and the burden of zero-dose and under-vaccinated children. By utilizing geolocated survey data from the 2023 ECV with geospatial covariates, we created 1 km × 1 km gridded coverage maps, validated our models at the cluster, provincial, and national levels, and quantified the number and distribution of children omitted by routine immunization services. The findings highlight the significance of geospatial modelling as a robust approach for identifying service delivery gaps and reaching underserved populations in countries like DRC, where routine data are often limited or unreliable, and conflict, geographic barriers, and weak infrastructure contribute to persistent inequities [41]. These findings are crucial for designing immunization strategies, especially in the context of the Immunization Agenda 2030 commitment to halve the number of zero-dose children worldwide by 2030 [42].

Our study identified substantial subnational variability in DTP1–3 and MCV1 vaccination coverage across DRC. This spatial heterogeneity aligns with trends in other LMIC’s, where remoteness, weak infrastructure and conflict-induced disruptions often lead to localized coverage gaps despite higher surrounding averages [26,31]. Consistent with these findings, recent spatial and multilevel analyses using the 2023–24 Demographic and Health Survey (DHS) demonstrate statistically significant clustering of full vaccination coverage (Moran’s I ≈ 0.4, *p* < 0.01), confirming that immunization outcomes in DRC are not randomly distributed but geographically structured [43]. In DRC, these disparities are further shaped by logistical challenges in accessing riverine and forested regions, shortages of the health workforce, and challenges in vaccine supply chains [15,19]. Spatial analyses also showed higher levels of full vaccination coverage in Kongo-Central, Kinshasa, and Kwango provinces, while lower coverage “cold spots” were concentrated in Mongala, Bas-Uele, Tshuapa, and Tshopo provinces [43]. The persistence of these geographic clusters suggests that spatial inequalities in vaccination coverage are linked to systematic differences in service delivery capacity rather than random variation [43].

The concentration of zero-dose children in certain health zones suggests that targeted microplanning could significantly enhance coverage without requiring nationwide interventions. Recent programmatic evidence from DRC supports the effectiveness of this strategy [44,45]. Geo-enabled microplans were effectively implemented, allowing health teams to more precisely map underserved areas, prioritize outreach routes, and track children who had previously been missed. Their implementation was linked to increased identification and immunization of zero-dose children, better planning of vaccine supply and delivery strategies, and more efficient use of limited resources. In addition, gender-responsive interventions strengthened women’s involvement in planning and service delivery, improved community outreach, and contributed to sustained gains in immunization coverage in the intervention provinces [44,46]. These findings highlight the importance of combining geospatial tools with participatory, gender-sensitive approaches to strengthen routine immunization services and reduce persistent coverage gaps. Beyond geographic factors, multilevel analyses highlights the importance of key socio-demographic and health service factors in influencing vaccination uptake [43]. Higher maternal education, greater household wealth, antenatal care utilization, and institutional delivery were all positively associated with full vaccination, whereas rural residence and long distance to health facilities remained significant barriers [43]. These findings suggest that spatial clusters of zero-dose children are often concentrated in socially and economically disadvantaged communities, reinforcing the need for integrated interventions that address both service delivery and demand-side barriers [43].

The increased dropout rates observed between DTP2 and DTP3, in contrast to those between DTP1 and DTP2, align with previous research findings from other sub-Saharan African countries. Earlier studies indicate that higher dropout rates later in the vaccination series are linked to caregiver fatigue, weak follow-up systems, and limitations in the health system’s capacity to identify and actively trace children who miss scheduled vaccination appointments [47,48,49]. The significantly elevated dropout rates in the low-coverage areas of Mongala, Maniema, and Tshopo suggest these regions face two major challenges: reaching underserved groups for the first dose and ensuring completion of the vaccination schedule once it has started. Addressing these gaps requires a combination of stronger community engagement to address hesitancy, mobile outreach to remote settlements, and improved follow-up systems to reduce missed subsequent doses. MCV1 coverage was consistently lower than DTP1–3 coverage in nearly all provinces, with especially large gaps observed in Mongala and Tshopo, where coverage in some areas fell below 20%. This pattern likely reflects a combination of poor geographic access, higher dropout rates later in the vaccination schedule, and weaker routine delivery of measles vaccination compared with infant DTP vaccination. These findings are especially concerning given the recurrent measles outbreaks reported in DRC, which have ranked among the highest globally in recent years [8,50]. The high proportion of MCV zero-dose children, over 60% in some provinces, highlights the urgent need to strengthen measles vaccination in underserved and hard-to-reach areas.

The Immunization Agenda 2030 goal of halving the prevalence of zero-dose children by 2030 is unlikely to be achieved in DRC without geographically targeted interventions. Over half of children under one in Mongala and Maniema have not received DTP1, underscores the magnitude of the situation. However, the high-resolution spatial outputs from this study provide practical insights to improve immunization strategies in the DRC. Mapping zero-dose and under-vaccinated children at fine spatial scales can support more targeted and efficient delivery of outreach services, vaccine supplies, and community mobilization activities. Dropout patterns between vaccine doses highlight the need for improved tracking systems, reminder-recall mechanisms, and health worker training to ensure completion of vaccination schedules. Our modelling demonstrates the potential of using geostatistical techniques with household survey data and high-resolution demographic inputs, to facilitate data-driven decision-making in complex and resource-limited settings such as DRC. Similar conclusions have been drawn from implementation research in DRC, where the integration of geospatial data into microplanning was found to enhance immunization coverage and equity outcomes [44,46].

This analysis is subject to several limitations that should be considered when interpreting the results. First, the primary data source for this work was the 2023 ECV survey, which is not an internationally recognized survey like the DHS, which were not available at the time of the study, typically used to develop modelled vaccination coverage estimates. The ECV data, available at the household level, offers significant opportunities for spatial granularity but also presents challenges due to irregularities that require data cleaning, particularly for household GPS coordinates. Additional inconsistencies were identified, including likely duplicate child records, necessitating the development of a systematic approach to detect and resolve them. Another limitation is the reliance on vaccination cards/documents and maternal or caregiver recall for vaccination status. In cases where vaccination cards were unavailable, recall may have been subject to error, potentially introducing information bias into the analysis. Estimating children under one year was challenging due to age heaping in datasets, requiring appropriate subnational age–sex distributions. We chose WorldPop Global projections for their complete administrative coverage, alignment with the UNWPP estimates, and transparent documentation of data inputs and methods. The modelling process was further complicated by the use of ratio indicators (DTP2/1 and DTP3/2) instead of directly modelling DTP2 and DTP3. Another limitation concerns the cross-validation strategy used to assess model predictive performance. We used random 10-fold cross-validation at the cluster level, in which observations were randomly partitioned into training and validation datasets. This approach does not explicitly account for spatial dependence and may result in geographically proximate clusters being represented in both the training and validation datasets, potentially leading to optimistic estimates of predictive performance. Spatially blocked k-fold cross-validation would provide a more stringent assessment of the model’s ability to predict in geographically unsampled areas. Future analysis incorporating spatially blocked cross-validation would further strengthen the assessment of out-of-sample predictive performance and the generalisability of the model to unsampled locations.

Temporal and age-group misalignment between the vaccination coverage and population datasets represents an additional limitation that may affect the estimated numbers of zero-dose and under-vaccinated children. Ideally, the vaccination coverage estimates and population denominators should correspond to the same reference year to ensure complete temporal consistency. However, while updated population estimates were available for 2024, equivalent nationally representative vaccination data were not available beyond the 2023 ECV survey during the time of analysis. We assumed that vaccination coverage did not change substantially between 2023 and 2024, given the relatively short one-year interval and the absence of a corresponding nationally representative coverage survey for 2024. This approach may introduce uncertainty because vaccination uptake may change over time and vaccination eligibility and uptake vary with age. The issue is particularly relevant for MCV1, as some children under one year may not have reached the recommended age for MCV1 vaccination. Consequently, the resulting estimates should be interpreted as approximate population-based projections rather than direct measurements of vaccination status among children under one year in 2024. In addition, geospatial covariates were derived from datasets spanning 2020 to 2024 because of data availability constraints, which may have introduced additional uncertainty into the modelling process. Future analyses incorporating temporally consistent vaccination coverage and population estimates would help reduce this uncertainty and improve the temporal comparability of the resulting estimates.

Despite these limitations, combining ECV data with geospatial covariates and population datasets enabled the generation of high-resolution estimates of vaccination coverage across the DRC. Future studies could further strengthen this approach by incorporating internationally standardised survey data, such as the recently released DRC DHS 2023 dataset, to support validation, calibration, and temporal comparisons of modelled estimates. Ongoing advancements in geospatial data quality, population modelling, and routine immunization reporting systems will also further enhance the reliability and programmatic utility of fine-scale vaccination coverage maps for immunization planning and decision making.

## 5. Conclusions

The analyses presented here offer critical insights into the nature of immunization gaps across the DRC, distinguishing between areas where access to the first dose is the primary challenge and those where follow-up and retention are the greatest barriers. The identification of high-burden zones for zero-dose and under-vaccinated children shows that incomplete vaccination remains a widespread and persistent public health concern, highlighting the need for targeted, evidence-based interventions tailored to the specific challenges identified. By combining household-level survey data with a suite of geospatial covariates and robust Bayesian modelling, this study demonstrates a practical method for generating granular coverage and zero-dose estimates that can be aggregated to any administrative level. These outputs provide valuable information for national and subnational immunization programs, enabling health managers to identify and prioritize areas with the highest burden of unvaccinated and under-vaccinated children. By embedding these insights into microplanning and integrating them with broader national and humanitarian strategies, there is significant potential to accelerate progress toward universal immunization coverage and support the Immunization Agenda 2030 goal of leaving no child behind.

## Figures and Tables

**Table 1:** Assessment of out-of-sample predictive performance using k-fold cross-validation.

Indicator	CORR	RMSE	MAE	AVG_BIAS
DTP1	0.760	0.146	0.108	0.004
DTP2/1	0.531	0.151	0.115	0.001
DTP3/2	0.496	0.188	0.140	−0.002
MCV1	0.657	0.195	0.156	0.002

**Table 2:** National level direct vs. modelled estimates for DTP1, DTP2, DTP3, and MCV1.

Source	DTP1 (%)	DTP2 (%)	DTP3 (%)	MCV1 (%)
2023 ECV Report (direct)	80.5	69.0	57.6	52.2
Modelled Estimate	81.1	71.8	60.3	52.7

## Data Availability

All the data used in the study are available from the sources referenced in the manuscript. The authors do not have the right to redistribute these data. The high-resolution modelled vaccination coverage estimates for DTP1–3 and MCV1, along with counts of zero-dose and under-vaccinated children in the Democratic Republic of Congo, have been published and are publicly available. These results can be accessed via WorldPop at the University of Southampton: Krishnaveni K.S., Utazi C.E., Cunningham A., Chamberlain H., Lazar A.N., Tatem A.J. (2025). Modelled gridded vaccination coverage and zero-dose and under-vaccinated children estimates for the Democratic Republic of Congo version 1.0 (2023). WorldPop, University of Southampton. doi: https://dx.doi.org/10.5258/SOTON/WP00878.

## Supplementary Materials

The following supporting information can be downloaded at https://www.mdpi.com/article/10.3390/vaccines14090754/s1, SI text. Supplementary file containing Tables S1–S3, Figures S1–S9, and additional text referenced in the manuscript (DOCX) [51,52,53,54,55,56].

## Abbreviations

The following abbreviations are used in this manuscript:

AIC	Akaike Information Criterion
DRC	Democratic Republic of the Congo
DTP	Diphtheria–tetanus–pertussis
EAF	Equity Accelerator Fund
GLM	Generalized Linear Models
INLA	Integrated Nested Laplace Approximation
KSPH	Kinshasa School of Public Health
LMIC	Low- and middle-income countries
MAE	Mean Absolute Error
OSM	OpenStreetMap
RMSE	Root Mean Square Error
SPDE	Stochastic Partial Differential Equation
VIF	Variance Inflation Factors
WHO	World Health Organization
