# Supplementary material for: High-Resolution Mapping of DTP1–3 and MCV1 Immunization Coverage and Estimates of Zero-Dose and Under-Vaccinated Children in the Democratic Republic of the Congo
Source: Vaccines (Basel). 2026 Aug 29;14(9):754. doi: 10.3390/vaccines14090754 (PMC13611382; doi:10.3390/vaccines14090754)
Supplement: Supplementary file 1 [file vaccines-14-00754-s001.zip › vaccines-4447453-supplementary.pdf]

vaccines

MDPI

Supplementary Information

This supplementary information accompanies the main manuscript and provides additional methodological details, results, and figures referenced in the main text.

1. Data Section
1.1 Data Cleaning
1.1.1 Correction of Inconsistencies in Data Relating to Individual Children
1.1.2 Correction of Erroneous Household Geolocations
1.2 Study Area
1.3 DTP1-3 and MCV1 Coverage Data
1.4 Geospatial Covariates
1.5 Under-1 Population Estimates
2. Results Section
2.1 Model Validation
2.2 Distribution of Administrative Units by Vaccination Coverage Level
2.3 High-Resolution Coverage Estimates
2.4 Burden of Zero-Dose Children

1. Data Section

1.1. Data Cleaning

The ECV2023 dataset required extensive cleaning to address inconsistencies in individual-level records and errors in geospatial data, ensuring the integrity of subsequent vaccination coverage analyses. Two primary dimensions of data quality were targeted: (1) inconsistencies in data relating to individual children, and (2) errors in household geolocations.

1.1.1 Correction of Inconsistencies in Data Relating to Individual Children

The ECV dataset is hierarchically structured using three levels of unique identifiers: households are identified by the HHS_KEY, mothers/guardians are identified by the MKEY, which incorporates the HHS_KEY of their household, and infants are identified by the CKEY, which incorporates the MKEY of their mother/guardian. Initial examination revealed inconsistencies in the combination of these keys, suggesting the presence of duplicated or mislinked records that likely referred to the same child. To resolve this, a new set of harmonized identifiers was created: HHS_KEY values were retained to maintain household-level continuity. A new mother/guardian ID (M_ID) was constructed using a concatenation of the HHS_KEY and the mother's date of birth. A new infant ID (ENFANT_ID) was generated by combining the M_ID with the child's sex, cleaned barcode, and date of birth of the infant. This restructuring facilitated the identification and removal of likely duplicate entries.

Prior to cleaning, the dataset contained 81,095 unique households, 81,937 unique mothers or guardians, and 83,414 unique children aged 6 to 23 months. Following the first step of data cleaning, which focused on correcting inconsistencies in records relating to individual children, the final analytical dataset comprised 80,951 households, 81,598 mothers or guardians, and 82,933 children. This step resulted in the removal of approximately 1.16% of household records, 0.41% of mother/guardian records, and 0.58% of child records.

1.1.2 Correction of Erroneous Household Geolocations

Spatial inconsistencies in household GPS coordinates were also addressed to ensure accurate mapping and spatial analysis. This was necessary because households were labelled as being within the same survey cluster, but with GPS coordinates indicating household locations in different health areas, with distances exceeding hundreds of kilometers in some cases. The cleaning process involved multiple steps:

1. Calculate the median household (HH) location (centroid) for each cluster of households. Then calculate the distance between each HH and its cluster centroid.
2. For each cluster, calculate the median absolute deviation (MAD) in HH-centroid distance. Calculate a distance threshold of MAD + 3*MAD and identify any HHs with a HH-centroid distance further than this threshold and mark those HHs as potential outliers.
3. Repeat steps (1) and (2) but for each cluster segment, in lieu of the whole cluster. Flag any HHs that are identified as potential outliers with BOTH cluster and segment thresholds (n=2,603).
4. Separately identify any remaining HHs for manual review where: (i) HH-centroid cluster distance is greater than 10km (rural) or 1.5km (urban) (n=1,922), or (ii) survey supervisor (q112 variable) is in subset with disproportionately high number of flagged HH locations (n=2,791).
5. Manually review the HH locations identified in step (4) and decide whether the whole cluster is dropped, or the HH location is updated.
6. For flagged HHs, update the location based on the nearest unflagged HH in the same cluster.

All flagged household locations were reviewed manually. For each, the research team decided whether to (i) exclude the cluster entirely or (ii) correct the household location using the nearest valid (unflagged) household location within the same cluster.

Following the final geolocation cleaning, which was conducted after correcting individual-level record inconsistencies, the number of survey units was updated from 2,712 clusters and 81,095 households (pre-cleaning) to 2,656 clusters and 79,404 households (post-cleaning), corresponding to the removal of 2.06% of clusters and 2.08% of household records. This comprehensive data cleaning ensured both the accuracy of household-level vaccination data and the validity of any subsequent spatial analyses or modelling efforts.

1.2 Study Area

The ECV survey was conducted across all 26 provinces as illustrated in Figure S1.

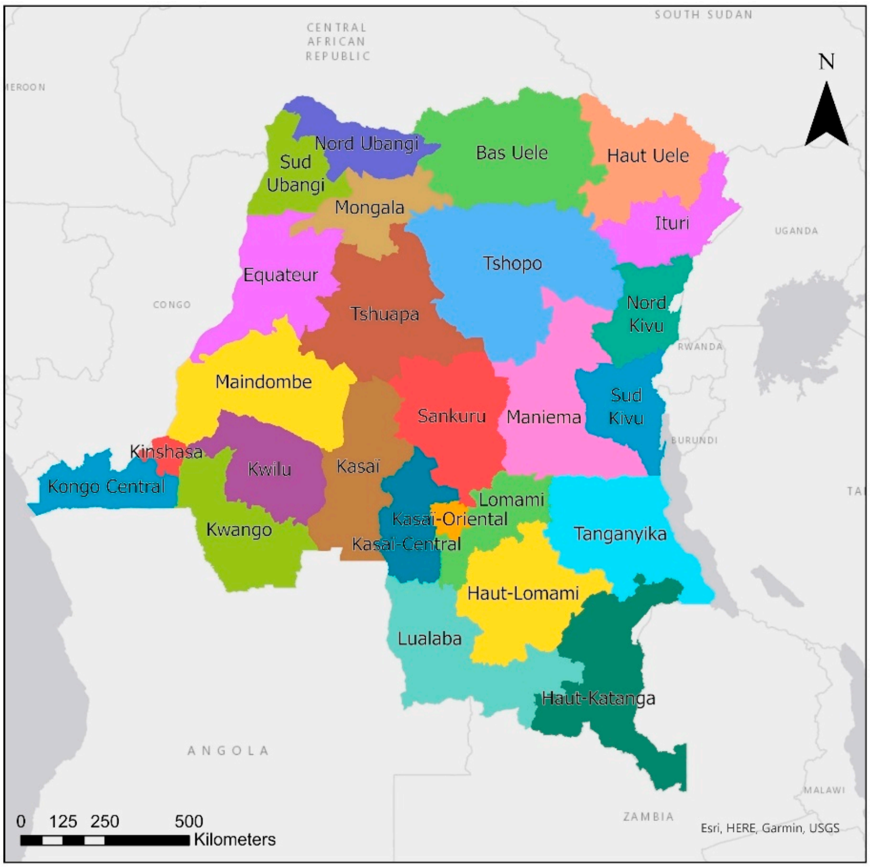

Figure S1: Relevant DRC administrative levels and subnational areas

1.3 DTP1-3 and MCV1 coverage data from the 2023 ECV data

Table S1: Age-Stratified Vaccination Coverage for Children Aged 6-23 Months: ECV 2023 Survey Data with Unweighted and Weighted Estimates

Age Group (Months)	Total Children	Vaccinated Children	Vaccination Percentage	Vaccination Percentage (Weighted)
		DTP1		
6-8	17773	12855	72.3	-
9-11	17112	13383	78.2	-
12-23	48042	37675	78.4	80.5
		DTP2		
6-8	17773	9948	55.9	-
9-11	17112	11026	64.4	-
12-23	48042	31830	66.2	69
		DTP3		
6-8	17773	7087	39.8	-
9-11	17112	8689	50.7	-
12-23	48042	26243	54.6	57.6
		MCV1		
6-8	17773	632	3.5	-
9-11	17112	5887	34.4	-
12-23	48042	24210	50.3	52.2

DTP1 coverage is initially high but declines markedly for DTP2 and DTP3, signifying a dropout in the immunisation schedule. The elevated dropout rate signifies problems in routine follow-up or service continuation. MCV1 coverage is the lowest among the four

antigens, indicating substantial gaps in measles vaccination and heightening the danger of outbreaks. The weighted national estimates are slightly greater than unweighted values (12–23 months).

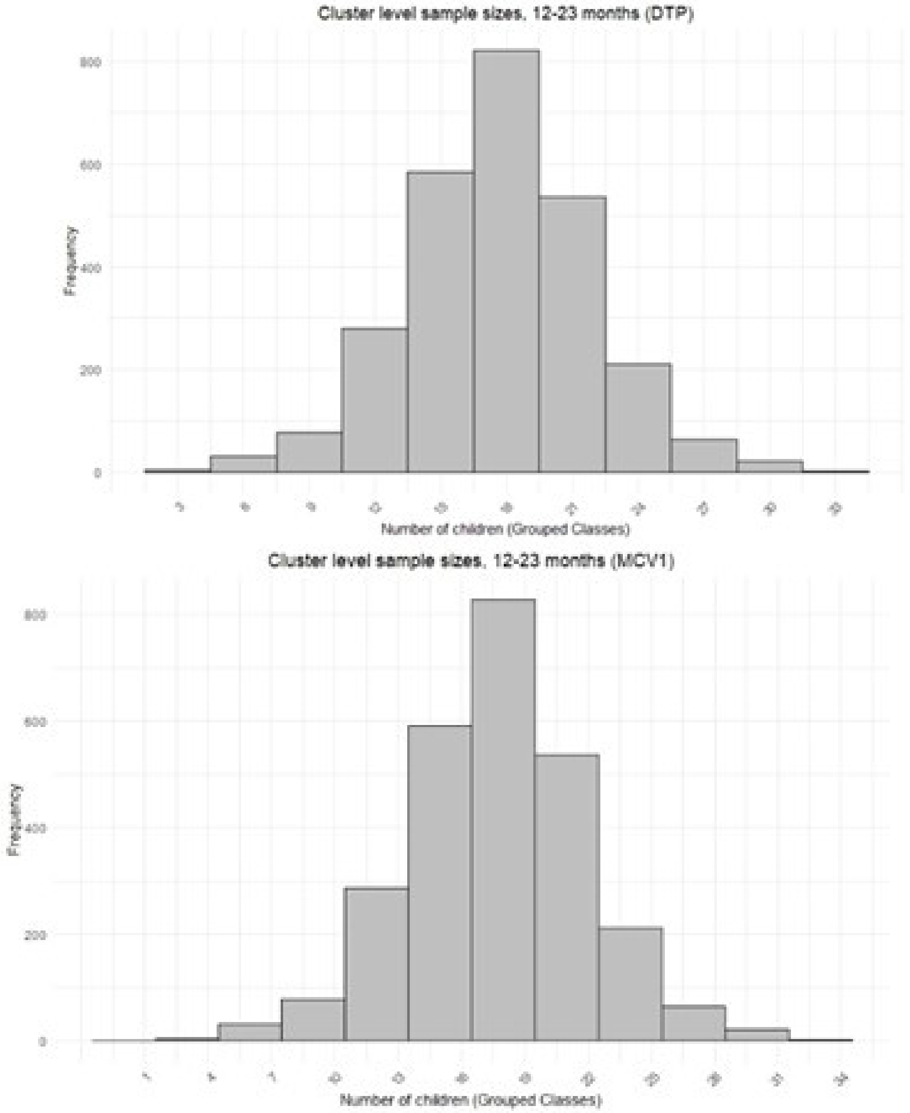

Figure S2: Distributions of sample sizes at the cluster level for children aged 12-23 months for DTP1-3 and MCV1 vaccine doses

Both histograms exhibit approximately normal distributions, with the majority of clusters containing between 20 and 60 children. The distribution for MCV1 (Figure 1) is slightly more peaked compared to DTP (Figure 2), suggesting a slightly higher frequency of clusters with mid-range sample sizes for MCV1. These distributions reflect adequate sample representation across clusters for both vaccines, which is important for generating robust coverage estimates.

1.4 Geospatial Covariates

Table S2: Details of geospatial covariates used in the modelling

	Name	Covariate Description	Year	Data Source
1.	Distance to coastline	Distance to open-water coastline	2023	Woods et al (2024) (51)
2	Distance to education facilities	Euclidean distance to education facilities, defined by OpenStreetMap per 100m	2023	OSMF (2024) (52)

	Name	Covariate Description	Year	Data Source
3	Distance to violence against civilians	Distance to violence against civilians conflict point events with filter applied to exclude data newer than 31/12/2022 to align with input survey data	2022	ACLED (2024) (53)
4	Distance to battles	Distance to battle conflict point events, with filter applied to exclude data newer than 31/12/2022 to align with input survey data	2022	ACLED (2024) (53)
5	Distance to protests	Distance to protest conflict point events, with filter applied to exclude data newer than 31/12/2022 to align with input survey data	2022	ACLED (2024) (53)
6	Distance to strategic and remote violence	Distance to strategic and remote violence conflict point events, with a filter applied to exclude data newer than 31/12/2022 to align with input survey data	2022	ACLED (2024) (53)
7	Distance to Herbaceous Cover, Grassland, Mosses edges	Distance to herbaceous cover, grassland, mosses edges. The values of the raster are from the cell centres to the nearest feature	2022	Woods et al (2024) (51)
8	Distance to Urban Areas edges	Distance to the edges of urban areas. The values of the raster are from the cell centres to the nearest feature	2022	Woods et al (2024) (51)
9	Near-infrared radiation reflectance 2022	Near-infrared radiation reflectance (unstated units) collated from monthly datasets into an annual total	2022	MODIS 'MOD13A3' Didan (2021) (54)
10	Malaria prevalence (latest year)	Proportion of Children 2 to 10 years of age showing, on a given year, detectable Plasmodium falciparum parasite 2000-2022	2022	Malaria Atlas Project Weiss et al (2019) (55)
11	Daily max temp 2021-2023	Daily max temperature measured as a monthly average in degrees Celsius, averaged across a 3-year period. (version 4.08)	2021-2023	CRU TS 4.08. https://crudata.uea.ac.uk/cru/data/hrg/#info Harris et al (2020) (56)
12	Distance to explosions and remote violence	Distance to explosions and remote violence	2022	ACLED (2024) (53)
13	Cattle density (average)	Cattle density per 10km pixel	2020	GLW 4: Gridded Livestock Density (Global - 2020 - 10 km), FAO (2020)
14	Distance to roads	Distance to roads. https://github.com/microsoft/RoadDetections	2023	Microsoft (2023)
15	Distance to Places of Worship	Euclidean distance to Places of worship, defined by OpenStreetMap per 100m	2023	OSMF (2024) (52)
16	Distance to shrubland edges	Distance to shrubland edges. The values of the raster are from the cell centres to the nearest feature	2022	Woods et al (2024) (51)

	Name	Covariate Description	Year	Data Source
17	Roads density	Road density. https://github.com/microsoft/RoadDetections	2023	WorldPop (Microsoft)
18	Proximity to protected areas (Category 1)	Distance to IUCN strict nature reserve and wilderness area edges 2015-2022	2022	Woods et al (2024) (51)
19	Walking Travel time to health facilities	Travel time based on only walking transport methods to the nearest Health Facility, as defined by the Malaria Atlas Project. The values of the raster are the from the cell centres to the nearest feature and are calculated in minutes	2020	Malaria Atlas Project: https://malariaatlas.org/research-project/accessibility-to-healthcare/ Weiss et al (2019) (55)
20	Rural/Urban stratification (1=urban, 2=rural)	GHS-SMOD classes 23 and 30 have been recoded to 1 (urban), with all remaining classes considered to be rural and recoded to have a value of 2	2024	Global Human Settlement - GHS-SMOD_GLOBE_R2023A - European Commission

* Note: References [51–56] are cited in the Supplementary Materials

1.5 Under-1 population estimates

To estimate the under-1 population across all provinces of the DRC, we created a mosaicked raster combining two complementary population datasets: the GRID3 v4.3 modelled population estimates (covering 15 provinces) (39) and the WorldPop Global2 gridded population estimates (37) (covering 11 provinces and adjusted to match the UN World Population Prospects 2024 national totals). Both input rasters had an approximate resolution of 100 meters. Estimates of under-1 children were generated by multiplying the total population in each raster cell by the under-1 derived from WorldPop Global2 demographic data (Figure S3).

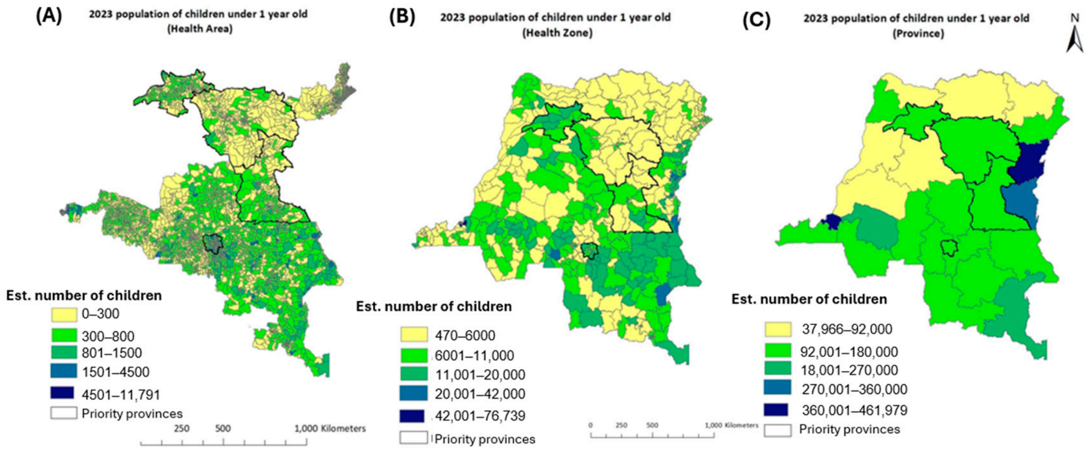

Figure S3: Population estimates for children aged under-1 aggregated to various administrative levels in the DRC. Panels A–C show population estimates at the health area (A), health zone (B), and provincial (C) levels.

The resulting mosaicked raster of under-1 population was then aggregated to a ~1 km resolution to spatially align with the modelled vaccination coverage surfaces. By multiplying these aligned population estimates with the grid-level coverage outputs, we derived the number of zero-dose children at the grid cell level. These counts were then summed to obtain estimates at the health area, health zone, and provincial levels.

2. Results Section

2.1 Model Validation

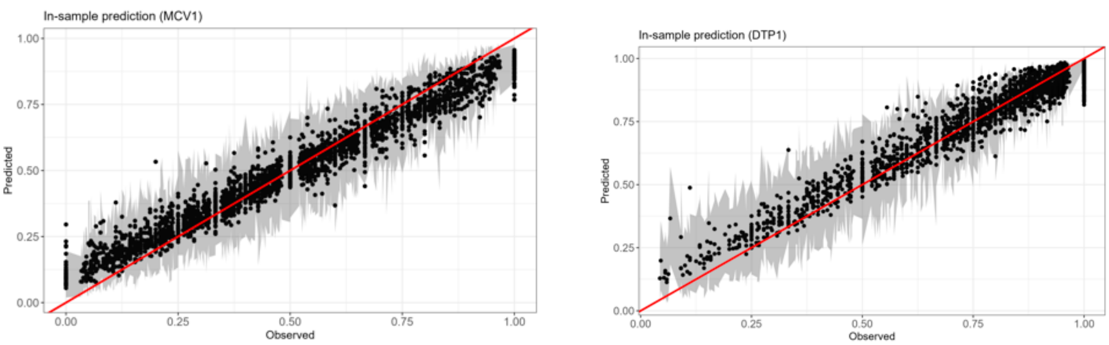

Figure S4: In-sample prediction plots for DTP1 and MCV1

2.2 Distribution of Administrative Units by Vaccination Coverage Level

Table S3: Distribution of administrative units in the Democratic Republic of Congo (DRC) by vaccination coverage level for DTP1-3 and MCV1

Admin Level	No. of Units	% vaccinated	DTP1 (n, %)	DTP2 (n, %)	DTP3 (n, %)	MCV1 (n, %)
Provinces	26	0 – 20	0(0%)	0(0%)	0(0%)	0(0%)
		20 – 40	0(0%)	2(7.7%)	6(23.1%)	8(30.8%)
		40 -60	5(19.2%)	5(19.2%)	9(34.6%)	15(57.7%)
		60 -80	8(30.8%)	11(42.3%)	10(38.5%)	2(7.7%)
		80 -100	13(50%)	8(30.8%)	1(3.8%)	1(3.8%)
Health zones	519	0 – 20	0(0%)	0(0%)	11(2.1%)	11(2.1%)
		20 – 40	14(2.7%)	47(9.1%)	89(17.1%)	141(27.2%)
		40 -60	67(12.9%)	97(18.7%)	175(33.7%)	234(45.1%)
		60 -80	124(23.9%)	201(38.7%)	170(32.8%)	97(18.7%)
		80 -100	314(60.5%)	174(3%)	74(14.3%)	36(6.9%)
Health areas (in 14 provinces)	5426	0 – 20	15(0.3%)	40(0.8%)	241(4.4%)	215(4%)
		20 – 40	319(5.9%)	694(12.8%)	1088(20.1%)	1682(31%)
		40 -60	881(16.2%)	1030(19%)	1580(29.1%)	2038(37.6%)
		60 -80	1152(21.2%)	1928(35.5%)	1705(31.4%)	1043(19.2%)
		80 -100	3059(56.4%)	1734(32%)	812(15%)	448(8.3%)
Health areas (in 4 priority provinces)	1330	0 – 20	15(1.1%)	39(2.9%)	191(14.4%)	208(15.6%)
		20 – 40	226(17%)	504(37.9%)	694(52.2%)	934(70.2%)
		40 -60	552(41.5%)	506(38%)	442(33.2%)	188(14.1%)
		60 -80	438(32.9%)	281(21.1%)	3(0.2%)	0(0%)
		80 -100	99(7.4%)	0(0%)	0(0%)	0(0%)

2.3 High-Resolution Coverage Estimates

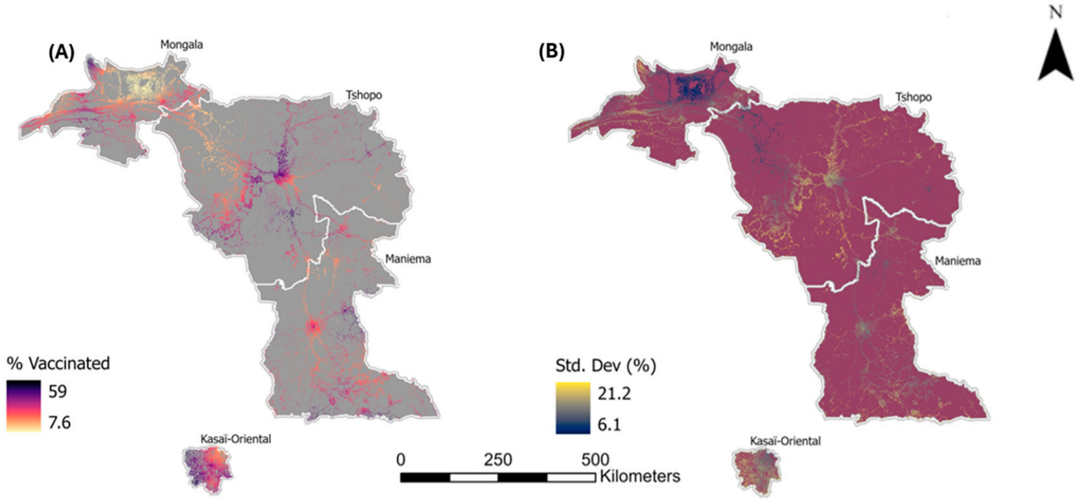

Figure S5: Constrained MCV1 coverage estimates at 1×1 km resolution for four provinces in the DRC. Panel A shows the estimated number of MCV1-vaccinated children aged 12–23 months, while Panel B shows the corresponding uncertainty in coverage, expressed as a percentage. These maps adjust spatial coverage estimates by population, illustrating that areas with low estimated vaccination coverage are frequently sparsely populated.

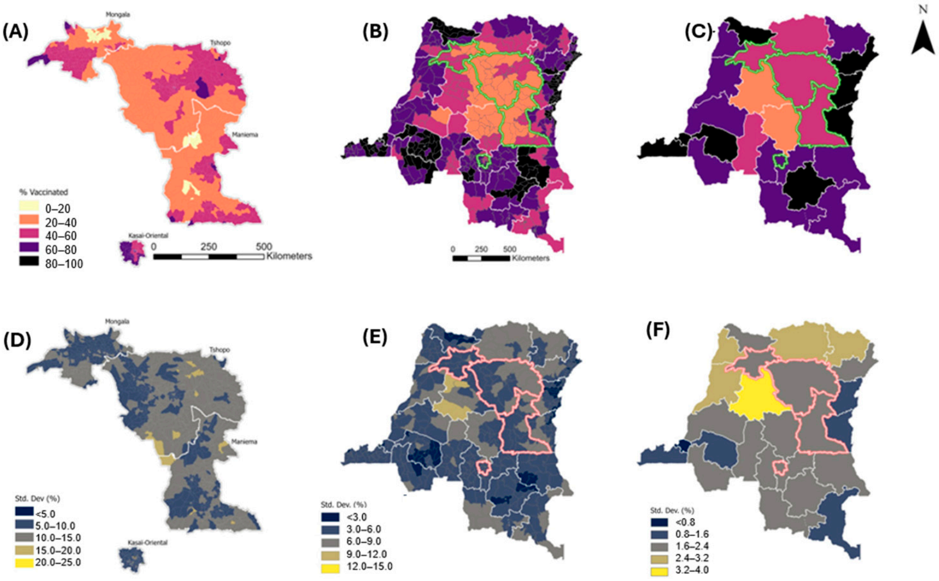

Figure S6: Estimated DTP2 coverage and associated uncertainty among children aged 12–23 months in the DRC, 2023.Panels A–C display estimated DTP2 vaccination coverage (%) at the health area (A), health zone (B), and provincial (C) levels. Panels D–F show the corresponding uncertainty in DTP2 coverage, expressed as the standard deviation (%), at the health area (D), health zone (E), and provincial (F) levels.

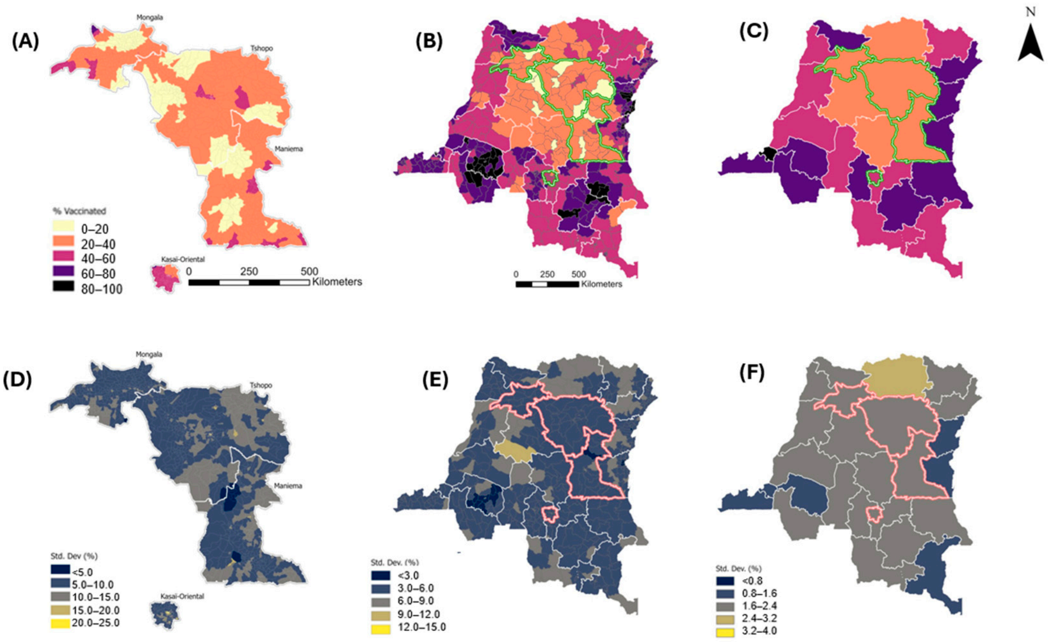

Figure S7: Estimated DTP3 coverage and associated uncertainty among children aged 12–23 months in the DRC, 2023.Panels A–C display estimated DTP3 vaccination coverage (%) at the health area (A), health zone (B), and provincial (C) levels. Panels D–F show the corresponding uncertainty in DTP3 coverage, expressed as the standard deviation (%), at the health area (D), health zone (E), and provincial (F) levels.

On a national scale, aggregating 1 km × 1 km grid-level estimates at the provincial level smooths out some of the fine-scale heterogeneity seen at the health zone and health area levels; however, significant inter-provincial differences in coverage remain apparent. Across the four study provinces, over 75% of health areas had DTP2 and DTP3 coverage below 60%, with only a few reaching moderate levels and none achieving 80% or higher. About 61% of health zones achieved at least 60% coverage for DTP2, but this proportion dropped to 47% for DTP3. Uncertainty around DTP3 estimates was generally lower than for DTP2, indicating more stable and consistent coverage patterns nationwide. At the provincial level, coverage patterns appeared more uniform, yet significant disparities persisted. Nearly three-quarters of provinces reached 60% or higher coverage for DTP2, but fewer than half achieved the same threshold for DTP3, with only one province exceeding 80% coverage. Similar to the pattern observed for DTP1, provinces in the southeast generally showed higher vaccination coverage, while several provinces in the central and western regions continued to report lower coverage, highlighting persistent geographic inequalities despite improvements at broader administrative levels.

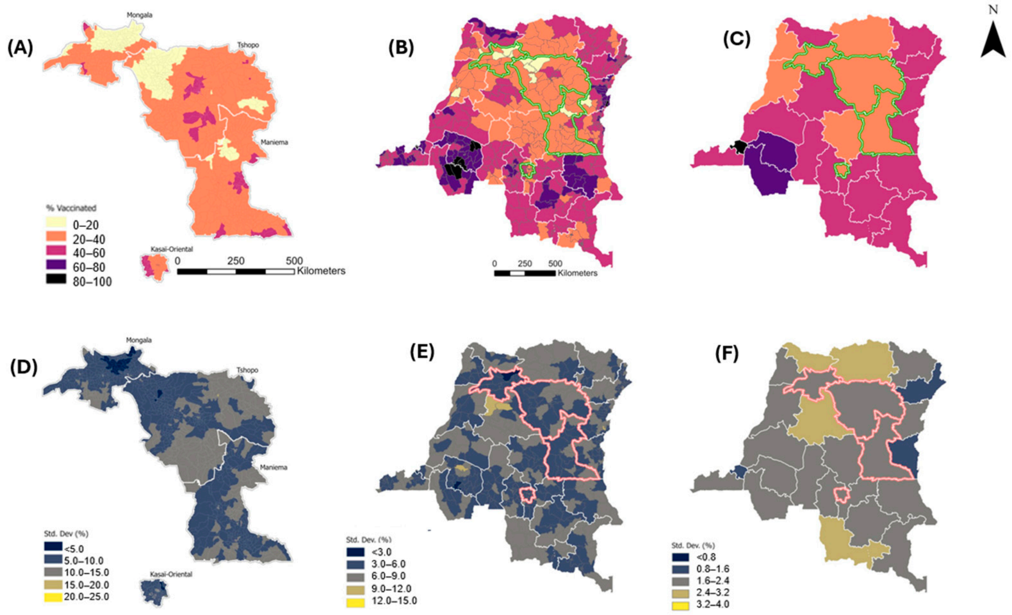

Figure S8: Estimated MCV1 coverage and associated uncertainty among children aged 12–23 months in the DRC, 2023.Panels A–C display estimated MCV1 vaccination coverage (%) at the health area (A), health zone (B), and provincial (C) levels. Panels D–F show the corresponding uncertainty in MCV1 coverage, expressed as the standard deviation (%), at the health area (D), health zone (E), and provincial (F) levels.

The coverage patterns were generally similar across all levels, with a significant portion of central DRC exhibiting coverage rates below 40%, and only a few areas reaching 80% or more. At the health area level (n = 1,330 across four priority provinces), MCV1 coverage was particularly low, with 85.8% of health areas reporting coverage below 40%. Only 14.1% achieved 40–60% coverage, and none exceeded 60%, indicating major local-level gaps in measles immunisation (Table S3 in SI text). At the health zone level (n = 519), coverage improved, but substantial variation persisted: 27.2% of zones achieved coverage of 20–40%, 45.1% achieved coverage of 40–60%, and only 6.9% reached coverage of 80% or more. Zones with very low coverage (<20%) were limited (2.1%), while those attaining moderate to high coverage (≥60%) were mainly concentrated in southern and western DRC. At the provincial level, only one province exceeded 80%. The lowest coverage (20–40%) was mainly confined to specific central and northern provinces, including Sud Ubangi, Équateur, Mongala, Bas-Uélé, Tshopo, Sankuru, and Maniema, highlighting persistent geographic pockets where MCV1 delivery remains weakest. Uncertainty in MCV1 estimates was generally low across all levels. Most health zones exhibited uncertainty below 6%, particularly in southern and western regions, suggesting robust and consistent data availability. Higher uncertainty (≥9%) was confined to scattered zones in north-central DRC, likely reflecting limited survey access or greater variability in reported data. At the provincial scale, overall uncertainty remained low, with most provinces below 2.4% and none reaching the highest uncertainty category. The relatively low variability across provinces suggests that aggregated estimates are stable and well-supported by underlying grid-level predictions, even in areas with low observed coverage.

2.4 Burden of Zero-Dose Children

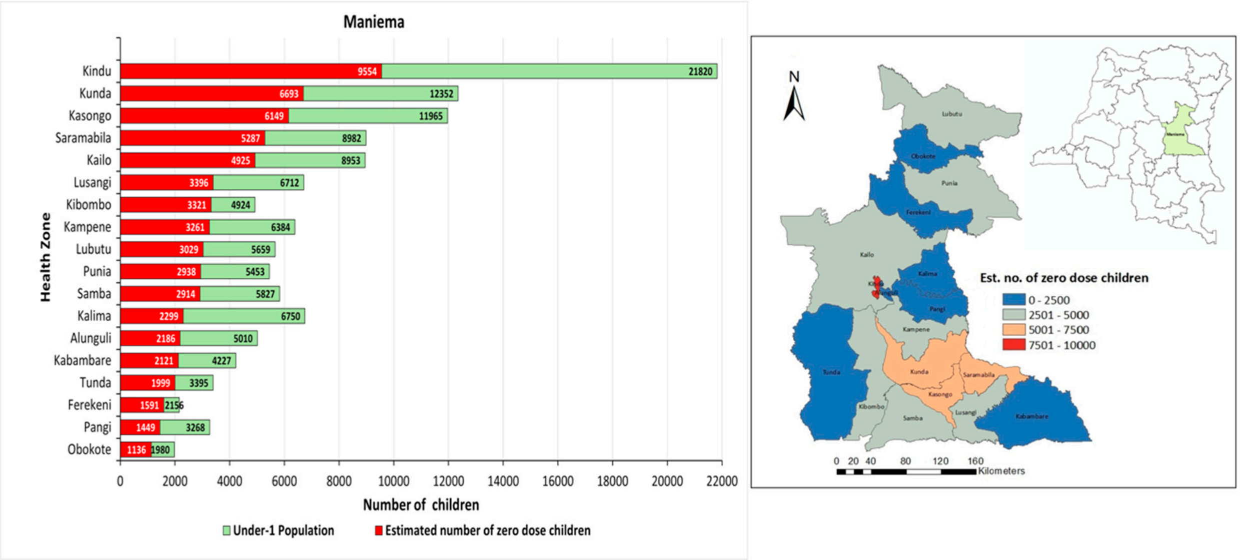

Figure S9: Spatial distribution and burden of zero-dose children in Maniema Province

For example, Kindu and Kunda emerge as the highest-burden zones, with 9,554 and 6,693 estimated DTP unvaccinated (zero-dose) children, respectively, among under-1 populations of 21,820 and 12,352, respectively. Other health zones with substantial burdens include Kasongo (6,149 zero-dose), Salamabila (5,287), and Kailo (4,925). Together, these five health zones account for more than half of all zero-dose children in the Maniema province. Conversely, health zones such as Obokote (1,136) and Pangi (1,449) contribute significantly fewer cases, despite having substantial populations under one year old, highlighting the geographic variability within the province.
